# Discrimination between Gaussian process models: active learning and static constructions

**DOI:** 10.1007/s00362-023-01436-x

**Published:** 2023-03-30

**Authors:** Elham Yousefi, Luc Pronzato, Markus Hainy, Werner G. Müller, Henry P. Wynn

**Affiliations:** 1 Institute of Applied Statistics, Johannes Kepler University, Altenberger Straße 69, 4040 Linz, Austria; 2 Université Côte d’Azur, CNRS, Laboratoire I3S - UMR 7271, 2000, route des Lucioles-Les Algorithmes-bât. Euclide B, 06900 Sophia Antipolis, France; 3 Department of Statistics, London School of Economics, Houghton Street, London, WC2A 2AE UK

**Keywords:** Model discrimination, Gaussian random field, Kriging, 62K05, 60G15

## Abstract

The paper covers the design and analysis of experiments to discriminate between two Gaussian process models with different covariance kernels, such as those widely used in computer experiments, kriging, sensor location and machine learning. Two frameworks are considered. First, we study sequential constructions, where successive design (observation) points are selected, either as additional points to an existing design or from the beginning of observation. The selection relies on the maximisation of the difference between the symmetric Kullback Leibler divergences for the two models, which depends on the observations, or on the mean squared error of both models, which does not. Then, we consider static criteria, such as the familiar log-likelihood ratios and the Fréchet distance between the covariance functions of the two models. Other distance-based criteria, simpler to compute than previous ones, are also introduced, for which, considering the framework of approximate design, a necessary condition for the optimality of a design measure is provided. The paper includes a study of the mathematical links between different criteria and numerical illustrations are provided.

## Introduction

The term ‘active learning’ [cf. Hino (2020) for a recent review] has replaced the traditional (sequential or adaptive) ‘design of experiments’ in the computer science literature, typically when the response is approximated by Gaussian process regression [GPR, cf. Sauer et al. (2022)]. It refers to selecting the most suitable inputs to achieve the maximum of information from the outputs, usually with the aim of improving prediction accuracy. A good overview is given in Chapter 6 of Gramacy (2020).

Frequently the aim of an experiment—in the broad sense of any data acquisition exercise—may rather be the discrimination between two or more potential explanatory models. When data can be sequentially collected during the experimental process, the literature goes back to the classic procedure of Hunter and Reiner (1965) and has generated ongoing research [see e.g. Schwaab et al. (2008), Olofsson et al. (2018) and Heirung et al. (2019)]. When the design needs to be fixed before the experiment and thus no intermediate data will be available, the literature is less developed. While in the classical (non)linear regression case the criterion of T-optimality [cf. Atkinson and Fedorov (1975)] and the numerous papers extending it was a major step, a similar breakthrough for Gaussian process regression is lacking.

With this paper we would like to investigate various sequential/adaptive and non-sequential design schemes for discriminating between the covariance structure of GPRs and their relative properties. When the observations associated with the already collected points are available, one may base the criterion on the predictions and prediction errors (Sect. 3.1). On the one hand, one natural choice will be to put the next design point where the symmetric Kullback–Leibler divergence between those two predictive (normal) distributions differs most. On the other hand, when the associated observations are not available, the incremental construction of the designs could be based on the mean squared error (MSE) for both models, assuming in turn that either of the two models is the true one (Sect. 3.2). We theoretically investigate the asymptotic differences of the criteria with respect to their discriminatory power.

The static construction of a set of optimal designs of given size for nominal model parameters is the last mode we have considered (Sect. 4). Our first choice is to use the difference between the expected values of the log likelihood ratios, assuming in turn that either of the two models is the true one. This is actually a function of the symmetric Kullback–Leibler divergence, which also arises from Bayesian considerations. In a similar spirit, the Fréchet distance between two covariance matrices provides another natural criterion. Some further novel but simple approaches are considered in this paper as well. In particular we are interested whether complex likelihood-based criteria like the Kullback–Leibler-divergence can be effectively replaced by simpler ones based directly on the respective covariance kernels. The construction of optimal design measures for model discrimination (approximate design theory) is considered in Sect. 5, where we investigate the geometric properties for some of the newly introduced criteria.

Eventually, to compare the discriminatory power of the resulting designs from different criteria, one can compute the correct classification (hit) rates after selecting the model with the higher likelihood value. In Sect. 6, a numerical illustration is provided for two Matérn kernels with different smoothness. Furthermore, we confirm the theoretical considerations about optimal design measures from Sect. 5 on a numerical example.

Except for adaptive designs, where the parameter estimates are continuously updated as new data arrive, we assume that the parameters of the models between which we want to discriminate are known. Therefore, our results are relevant in situations where there is strong prior knowledge about the possible models, for example through previously collected data.

## Notation

One of the most popular design criteria for discriminating between rival models is T-optimality (Atkinson and Fedorov 1975). This criterion is only applicable when the observations are independent and normally distributed with a constant variance. López-Fidalgo et al. (2007) generalised the normality assumption and developed an optimal discriminating design criterion to choose among non-normal models. The criterion is based on the log-likelihood ratio test under the assumption of independent observations. We denote by $\varphi _0(y,x,\theta _0)$ and $\varphi _1(y,x,\theta _1)$ the two rival probability density functions for one observation *y* at point *x*. The following system of hypotheses might be considered:

$$\begin{aligned}{} & {} H_0:\varphi (y,x)=\varphi _0(y,x,\theta _0)\\{} & {} H_1:\varphi (y,x)=\varphi _1(y,x,\theta _1) \end{aligned}$$

where $\varphi _1(y,x,\theta _1)$ is assumed to be the true model. A common test statistic is the log-likelihood ratio given as

$$\begin{aligned} L=-\log \dfrac{\varphi _0(y,x,\theta _0)}{\varphi _1(y,x,\theta _1)}=\log \dfrac{\varphi _1(y,x,\theta _1)}{\varphi _0(y,x,\theta _0)}, \end{aligned}$$

where the null hypothesis is rejected when $\varphi _1(y,x,\theta _1)>\varphi _0(y,x,\theta _0)$ or equivalently when $L>0$. The power of the test refers to the expected value of the log-likelihood ratio criterion under the alternative hypothesis $H_1$. We have

1 $$\begin{aligned} {\textsf{E}}_{H_1}(L)={\textsf{E}}_{1}(L)= & {} \int \varphi _1(y,x,\theta _1)\log \left\{ \dfrac{\varphi _1(y,x,\theta _1)}{\varphi _0(y,x,\theta _0)}\right\} \textrm{d}y\nonumber \\= & {} D_{KL}(\varphi _1\Vert \varphi _0), \end{aligned}$$

where $D_{KL}(\varphi _1\Vert \varphi _0)$ is the Kullback–Leibler distance between the true and the alternative model (Kullback and Leibler 1951).

Interchanging the two models in the null and the alternative hypothesis, the power of the test would be

2 $$\begin{aligned} {\textsf{E}}_{0}(-L)= & {} D_{KL}(\varphi _0\Vert \varphi _1). \end{aligned}$$

If it is not clear in advance which of the two models is the true model, one might consider to search for a design optimising a convex combination of (1) and (2), most commonly using weights 1/2 for each model. This would be equivalent to maximising the symmetric Kullback–Leibler distance

$$\begin{aligned} D_{KL}(\varphi _0, \varphi _1)= & {} \frac{1}{2} \left[ D_{KL}\left( \varphi _0\Vert \varphi _1\right) + D_{KL}\left( \varphi _1\Vert \varphi _0\right) \right] . \end{aligned}$$

In this paper we will consider random fields, i.e. we will allow for correlated observations. As we assume that the mean function is known and the same for all models, without loss of generality we can set the mean function equal 0 everywhere. We are solely concerned with discriminating with respect to the covariance structure of the random fields. When the random fields are Gaussian, we might still base the design strategy on the log-likelihood ratio criterion to choose among two rival models.

For a positive definite kernel $K(x,x')$ and an *n*-point design ${\textbf{X}}_n=(x_1,\ldots ,x_n)$, ${\textbf{k}}_n(x)$ is the *n*-dimensional vector $(K(x,x_1),\ldots ,K(x,x_n))^\top$ and ${\textbf{K}}_n$ is the $n\times n$ (kernel) matrix with elements $\{{\textbf{K}}_n\}_{i,j}=K(x_i,x_j)$. Although *x* is not bold, it may correspond to a point in a (compact) set ${{\mathscr {X}}}\subset {\mathbb {R}}^d$. Assume that *Y*(*x*) corresponds to the realisation of a random field $Z_x$, indexed by *x* in ${{\mathscr {X}}}$, with zero mean ${\textsf{E}}\{Z_x\}=0$ for all *x* and covariance ${\textsf{E}}\{Z_x Z_{x'}\}=K(x,x')$ for all $(x,x')\in {{\mathscr {X}}}^2$. Our prediction of a future observation *Y*(*x*) based on observations ${\textbf{Y}}_n=(Y(x_1),\ldots ,Y(x_n))^\top$ corresponds to the best linear unbiased predictor (BLUP) ${{\widehat{\eta }}}_n(x)={\textbf{k}}_n^\top (x){\textbf{K}}_n^{-1}{\textbf{Y}}_n$. The associated prediction error is $e_n(x)=Y(x)-{{\widehat{\eta }}}_n(x)$ and we have

$$\begin{aligned} {\textsf{E}}\left\{ e_n^2(x)\right\} = \rho _n^2(x) = K(x,x)-{\textbf{k}}_n^\top (x){\textbf{K}}_n^{-1}{\textbf{k}}_n(x). \end{aligned}$$

The index *n* will often be omitted when there is no ambiguity, and in that case ${\textbf{k}}_i(x)={\textbf{k}}_{n,i}(x)$, ${\textbf{K}}_i={\textbf{K}}_{n,i}$, $e_i(x)=e_{n,i}(x)$, $\rho _i^2(x)=\rho _{n,i}^2(x)$ will refer instead to model *i*, with $i\in \{0,1\}$. We shall need to distinguish between the cases where the truth is model 0 or model 1, and following Stein (1999, p. 58) we denote by ${\textsf{E}}_i$ the expectation computed with model *i* assumed to be true. We reserve the notation $\rho _i^2(x)$ to the case where the expectation is computed with the true model; i.e.,

$$\begin{aligned} \rho _i^2(x) = {\textsf{E}}_i \left\{ e_i^2(x)\right\} . \end{aligned}$$

Hence we have $\rho _0^2(x) = {\textsf{E}}_0\{e_0^2(x)\}= K_0(x,x)-{\textbf{k}}_0^\top (x){\textbf{K}}_0^{-1}{\textbf{k}}_0(x)$ and calculation gives

3 $$\begin{aligned}{} & {} {\textsf{E}}_0\{e_1^2(x)\} = K_0(x,x)+{\textbf{k}}_1^\top (x){\textbf{K}}_1^{-1}{\textbf{K}}_0{\textbf{K}}_1^{-1}{\textbf{k}}_1(x)- 2\,{\textbf{k}}_1^\top (x){\textbf{K}}_1^{-1}{\textbf{k}}_0(x) , \nonumber \\{} & {} {\textsf{E}}_0\{[e_1(x)-e_0(x)]^2\} = {\textsf{E}}_0\{e_1^2(x)\} - {\textsf{E}}_0\{e_0^2(x)\} , \end{aligned}$$

with an obvious permutation of indices 0 and 1 when assuming the model 1 is true to compute ${\textsf{E}}_1\{\cdot \}$.

If model 0 is correct, the prediction error is larger when we use model 1 for prediction than if we use the BLUP (i.e., model 0). Stein (1999, p. 58) shows that the relation

$$\begin{aligned} \frac{{\textsf{E}}_0\left\{ e_1^2(x)\right\} }{{\textsf{E}}_0\left\{ e_0^2(x)\right\} } = 1+ \frac{{\textsf{E}}_0\left\{ [e_1(x)-e_0(x)]^2\right\} }{{\textsf{E}}_0\left\{ e_0^2(x)\right\} } \end{aligned}$$

shown above is valid more generally for models with linear trends.

Also of interest is the assumed mean squared error (MSE) ${\textsf{E}}_1\{e_1^2(x)\}$ when we use model 1 for assessing the prediction error (because we think it is correct) while the truth is model 0, and in particular the ratio

$$\begin{aligned} \frac{{\textsf{E}}_1\left\{ e_1^2(x)\right\} }{{\textsf{E}}_0\left\{ e_1^2(x)\right\} } = \frac{K_1(x,x)-{\textbf{k}}_1^\top (x){\textbf{K}}_1^{-1}{\textbf{k}}_1(x)}{{\textsf{E}}_0\left\{ e_1^2(x)\right\} } , \end{aligned}$$

which may be larger or smaller than one.

Another important issue concerns the choice of covariance parameters in $K_0$ and $K_1$. Denote $K_i(x,x')=\sigma _i^2\,C_{i,\theta _i}(x,x')$, $i=0,1$, $(x,x')\in {{\mathscr {X}}}^2$, where the $\sigma _i^2$ define the variance, the $\theta _i$ may correspond to correlation lengths in a translation invariant model and are thus scalar in the isotropic case, and $C(x,x')$ defines a correlation.

## Prediction-based discrimination

For the incremental construction of a design for model discrimination, points are added conditionally on previous design points. We can distinguish the case where the observations associated with those previous points are available and can thus be used to construct a sequence of predictions (sequential, i.e., conditional, construction) from the unconditional case where observations are not used.

### Sequential (conditional) design

Consider stage *n*, where *n* design points ${\textbf{X}}_n$ and *n* observations ${\textbf{Y}}_n$ are available. Assuming that the random field is Gaussian, when model *i* is true we have $Y(x) \sim {{\mathscr {N}}}({{\widehat{\eta }}}_{n,i}(x),\rho _{n,i}^2(x))$. A rather natural choice is to choose the next design point $x_{n+1}$ where the symmetric Kullback–Leibler divergence between those two normal distributions differs most; that is,

4 $$\begin{aligned} x_{n+1}\in & {} \textrm{Arg}\max _{x\in {{\mathscr {X}}}} \frac{\rho _{n,0}^2(x)}{\rho _{n,1}^2(x)} + \frac{\rho _{n,1}^2(x)}{\rho _{n,0}^2(x)} + [{{\widehat{\eta }}}_{n,1}(x)-{{\widehat{\eta }}}_{n,0}(x)]^2\, \left[ \frac{1}{\rho _{n,0}^2(x)}+ \frac{1}{\rho _{n,1}^2(x)} \right] .\nonumber \\ \end{aligned}$$

Other variants could be considered as well, such as

$$\begin{aligned} x_{n+1}\in & {} \textrm{Arg}\max _{x\in {{\mathscr {X}}}}\, [{{\widehat{\eta }}}_{n,1}(x)-{{\widehat{\eta }}}_{n,0}(x)]^2 , \\ x_{n+1}\in & {} \textrm{Arg}\max _{x\in {{\mathscr {X}}}}\, \frac{[{{\widehat{\eta }}}_{n,1}(x)-{{\widehat{\eta }}}_{n,0}(x)]^2}{\rho _{n,0}^2(x)+\rho _{n,1}^2(x)} , \\ x_{n+1}\in & {} \textrm{Arg}\max _{x\in {{\mathscr {X}}}}\, [{{\widehat{\eta }}}_{n,1}(x)-{{\widehat{\eta }}}_{n,0}(x)]^2\, \left[ \frac{1}{\rho _{n,0}^2(x)}+ \frac{1}{\rho _{n,1}^2(x)} \right] . \end{aligned}$$

They will not be considered in the rest of the paper.

If necessary one can use plug-in estimates ${{\widehat{\sigma }}}_{n,i}^2$ and ${{\widehat{\theta }}}_{n,i}$ of $\sigma _i^2$ and $\theta _i$, for instance maximum likelihood (ML) or leave-one-out estimates based on ${\textbf{X}}_n$ and ${\textbf{Y}}_n$, when we choose $x_{n+1}$. Note that the value of $\sigma ^2$ does not affect the BLUP ${{\widehat{\eta }}}_n(x)={\textbf{k}}_n^\top {\textbf{K}}_n^{-1}{\textbf{Y}}_n$. In the paper we do not address the issues related to the estimation of $\sigma ^2$ or of the correlation length or smoothness parameters of the kernel; one may refer to Karvonen et al. (2020) and the recent papers Karvonen (2022), Karvonen and Oates (2022) for a detailed investigation. The connection between the notion of microergodicity, related to the consistency of the maximum-likelihood estimator, and discrimination through a KL divergence criterion is nevertheless considered in Example 1 below.

### Incremental (unconditional) design

Consider stage *n*, where *n* design points ${\textbf{X}}_n$ are available. We base the choice of the next point on the difference between the MSEs for both models, assuming that one or the other is true. For instance, assuming that model 0 is true, the difference between the MSEs is ${\textsf{E}}_0\{e_1^2(x)\}-{\textsf{E}}_0\{e_0^2(x)\}={\textsf{E}}_0\{[e_1(x)-e_0(x)]^2\}={\textsf{E}}_0 \{[{{\widehat{\eta }}}_{n,1}(x)-{{\widehat{\eta }}}_{n,0}(x)]^2\}$.

A first, un-normalised, version is thus

5 $$\begin{aligned} \phi _A(x)= & {} {\textsf{E}}_0\left\{ [e_1(x)-e_0(x)]^2\right\} + {\textsf{E}}_1\left\{ [e_1(x)-e_0(x)]^2\right\} \, \nonumber \\= & {} {\textsf{E}}_0\left\{ e_1^2(x)\right\} - {\textsf{E}}_0\left\{ e_0^2(x)\right\} + {\textsf{E}}_1\left\{ e_0^2(x)\right\} -{\textsf{E}}_1\left\{ e_1^2(x)\right\} . \end{aligned}$$

A normalisation seems in order here too, such as

6 $$\begin{aligned} \phi _B(x){} & {} =\frac{{\textsf{E}}_0\left\{ [e_1(x)-e_0(x)]^2\right\} }{\rho _{n,0}^2(x)} + \frac{{\textsf{E}}_1\left\{ [e_1(x)-e_0(x)]^2\right\} }{\rho _{n,1}^2(x)}\nonumber \\{} & {} = \frac{{\textsf{E}}_0\left\{ e_1^2(x)\right\} }{{\textsf{E}}_0\left\{ e_0^2(x)\right\} } + \frac{{\textsf{E}}_1\left\{ e_0^2(x)\right\} }{{\textsf{E}}_1\left\{ e_1^2(x)\right\} } - 2. \end{aligned}$$

A third criterion is based on the variation of the symmetric Kullback-Leibler divergence (10) of Sect. 4 when adding an $(n+1)$-th point *x* to ${\textbf{X}}_n$. Direct calculation, using

$$\begin{aligned} {\textbf{K}}_{n+1,i}=\left( \begin{array}{cc} {\textbf{K}}_{n,i} &{} {\textbf{k}}_{n,i}(x) \\ {\textbf{k}}_{n,i}^\top (x) &{} K_i(x,x) \\ \end{array} \right) ,\quad \ i=0,1, \end{aligned}$$

and the expression of the inverse of a block matrix, gives

$$\begin{aligned} \Phi _{KL\,[K_{0},K_{1}]}({\textbf{X}}_n\cup \{x\}) = \Phi _{KL\,[K_{0},K_{1}]}({\textbf{X}}_n) + \frac{1}{2}\,\left[ \frac{{\textsf{E}}_1\{e_0^2(x)\}}{{\textsf{E}}_0\{e_0^2(x)\}} + \frac{{\textsf{E}}_0\{e_1^2(x)\}}{{\textsf{E}}_1\{e_1^2(x)\}}\right] -1 . \end{aligned}$$

We thus define

7 $$\begin{aligned} \phi _{KL}(x)=\frac{1}{2}\,\left[ \frac{{\textsf{E}}_1\{e_0^2(x)\}}{{\textsf{E}}_0\{e_0^2(x)\}} + \frac{{\textsf{E}}_0\{e_1^2(x)\}}{{\textsf{E}}_1\{e_1^2(x)\}}\right] -1, \end{aligned}$$

to be maximised with respect to $x\in {{\mathscr {X}}}$.

Although the $\sigma _i^2$ do not affect predictions, ${\textsf{E}}_i\{e_j^2(x)\}$ is proportional to $\sigma _i^2$. Unless specific information is available, it seems reasonable to assume that $\sigma _0^2=\sigma _1^2=1$. Other parameters $\theta _i$ should be chosen to make the two kernels the most similar, which seems easier to consider in the approach presented in Sect. 4, see (11). In the rest of this section we suppose that the parameters of both kernels are fixed.

The un-normalised version $\phi _A(x)$ given by (5) could be used to derive a one-step (non-incremental) criterion, in the same spirit as those of Sect. 4, through integration with respect to *x* for a given measure $\mu$ on ${{\mathscr {X}}}$. Indeed, we have

$$\begin{aligned}&{\textsf{E}}_0\left\{ [e_1(x)-e_0(x)]^2\right\} \\ {}&\quad = {\textbf{k}}_0^\top (x){\textbf{K}}_0^{-1}{\textbf{k}}_0({\textbf{x}})+ {\textbf{k}}_1^\top (x){\textbf{K}}_1^{-1}{\textbf{K}}_0{\textbf{K}}_1^{-1}{\textbf{k}}_1(x)-2\,{\textbf{k}}_1^\top (x){\textbf{K}}_1^{-1}{\textbf{k}}_0(x), \end{aligned}$$

so that

$$\begin{aligned}&\int _{{\mathscr {X}}}{\textsf{E}}_0\left\{ [e_1(x)-e_0(x)]^2\right\} \, \textrm{d}\mu (x) \\&\quad =\textrm{trace}\left[ {\textbf{K}}_0^{-1}{\textbf{A}}_0(\mu )+{\textbf{K}}_1^{-1}{\textbf{K}}_0{\textbf{K}}_1^{-1}{\textbf{A}}_1(\mu )-2\,{\textbf{K}}_1^{-1}{\textbf{A}}_{0,1}(\mu ) \right] \,, \end{aligned}$$

where ${\textbf{A}}_i(\mu )=\int _{{\mathscr {X}}}{\textbf{k}}_i(x){\textbf{k}}_i^\top (x)\, \textrm{d}\mu (x)$, $i=0,1$, and ${\textbf{A}}_{0,1}(\mu )=\int _{{\mathscr {X}}}{\textbf{k}}_0(x) {\textbf{k}}_1^\top (x)\, \textrm{d}\mu (x)$. Similarly,

$$\begin{aligned}&\int _{{\mathscr {X}}}{\textsf{E}}_1\left\{ [e_1(x)-e_0(x)]^2\right\} \, \textrm{d}\mu (x) \\&\quad =\textrm{trace}\left[ {\textbf{K}}_1^{-1}{\textbf{A}}_1(\mu )+{\textbf{K}}_0^{-1}{\textbf{K}}_1{\textbf{K}}_0^{-1}{\textbf{A}}_0(\mu )-2\,{\textbf{K}}_0^{-1}{\textbf{A}}_{0,1}(\mu ) \right] \,. \end{aligned}$$

The matrices ${\textbf{A}}_i(\mu )$ and ${\textbf{A}}_{0,1}(\mu )$ can be calculated explicitly for some kernels and measures $\mu$. This happens in particular when ${{\mathscr {X}}}=[0,1]^d$, the two kernels $K_i$ are separable, i.e., products of one-dimensional kernels on [0, 1], and $\mu$ is uniform on ${{\mathscr {X}}}$.

#### Example 1: exponential covariance, no microergodic parameters

We consider Example 6 in Stein (1999, p. 74) and take $K_i(x,x')={\textsf{e}}^{-\alpha _i |x-x' |}/\alpha _i$, $i=0,1$. The example focuses on two difficulties: first, the two kernels only differ by their parameter values; second, the particular relation between the variance and correlation length makes the parameters $\alpha _i$ not microergodic and they cannot be estimated consistently from observations on a bounded interval; see Stein (1999, Chap. 6). It is interesting to investigate the behaviour of the criteria (5), (6) and (7) in this particular situation.

We suppose that *n* observations are made at $x_i=(i-1)/(n-1)$, $i=1,\ldots ,n\ge 2$. We denote $\delta =\delta _n=1/[2(n-1)]$ the half-distance between two design points. The particular Markovian property of random processes with kernels $K_i$ simplifies the analysis. The prediction and MSE at a given $x\in (0,1)$ only depend on the position of *x* relative to its two closest neighbouring design points; moreover, all other points have no influence. Therefore, due to the regular repartition of the $x_i$, we only need to consider the behaviour in one (any) interval ${\mathbb {I}}_i=[a_i,b_i]=[x_i,x_{i+1}]$.

We always have $\phi _A(x) \rightarrow 0$ as $x\rightarrow x_i\in {{\mathscr {X}}}_n$. Numerical calculation shows that for $\delta _n$ small enough, $\phi _A(\cdot )$ has a unique maximum in ${\mathbb {I}}_i$ at the centre $C_i=(x_i+x_{i+1})/2$. The next design point $x_{n+1}$ that maximises $\phi _A(\cdot )$ is then taken at $C_i$ for one of the $n-1$ intervals, and we get

$$\begin{aligned} \phi _A(C_i)= \frac{1}{4}\, \frac{(\alpha _1-\alpha _0)^2(\alpha _1+\alpha _0)^3}{\alpha _0\alpha _1} \, \delta _n^4+ {\mathcal {O}}(\delta _n^5) , \ n\rightarrow \infty . \end{aligned}$$

Similar results apply to the case where the design ${\textbf{X}}_n$ contains the endpoints 0 and 1 and its covering radius $\textsf{CR}({\textbf{X}}_n)=\max _{x\in [0,1]}\min _{i=1,\ldots ,n} |x-x_i |$ tends to zero, the points $x_i$ being not necessarily equally spaced: $C_i$ is then the centre of the largest interval $[x_i,x_{i+1}]$ and $\delta _n=\textsf{CR}({\textbf{X}}_n)$.

When $\delta _n$ is large compared to the correlation lengths $1/\alpha _0$ and $1/\alpha _1$, there exist two maxima, symmetric with respect to $C_i$, that get closer to the extremities of ${\mathbb {I}}_i$ as $\alpha _1$ increases, and $C_i$ corresponds to a local minimum of $\phi _A(\cdot )$. This happens for instance when $\alpha _0\,\delta _n=1$ and $\alpha _1\,\delta _n \gtrsim 2.600455$.

A similar behaviour is observed for $\phi _B(x)$ and $\phi _{KL}(x)$: for small enough $\delta _n$ they both have a unique maximum in ${\mathbb {I}}_i$ at $C_i$, with now

$$\begin{aligned}{} & {} \phi _B(C_i) = \frac{1}{4}\, \frac{(\alpha _1-\alpha _0)^2(\alpha _1+\alpha _0)^3}{\alpha _0\alpha _1} \, \delta _n^3+ {\mathcal {O}}(\delta _n^4) , \ n\rightarrow \infty ,\\{} & {} \phi _{KL}(C_i) = \frac{1}{8}\, \frac{(\alpha _1-\alpha _0)^2(\alpha _1+\alpha _0)^3}{\alpha _0\alpha _1} \, \delta _n^3+ {\mathcal {O}}(\delta _n^4) , \ n\rightarrow \infty . \end{aligned}$$

Also, $\phi _B(x) \rightarrow 0$ and $\phi _{KL}(x) \rightarrow 0$ as $x\rightarrow x_i\in {\textbf{X}}_n$. For large values of $\delta _n$ compared to the correlation lengths $1/\alpha _0$ and $1/\alpha _1$, there exist two maxima in ${\mathbb {I}}_i$, symmetric with respect to $C_i$. When $\alpha _0\,\delta _n=1$, this happens for instance when $\alpha _1\,\delta _n \gtrsim 2.020178$ for $\phi _B(\cdot )$ and when $\alpha _1\,\delta _n \gtrsim 7.251623$ for $\phi _{KL}(\cdot )$. However, in the second case the function is practically flat between the two maxima.

The left panel of Fig.  presents $\phi _A(x)$, $\phi _B(x)$ and $\phi _{KL}(x)$ for $x\in [x_1,x_2]=[0,0.1]$ when $n=11$ ($\delta _n= 0.05$) and $\alpha _0=1$, $\alpha _1=10$. The right panel is for $\alpha _0\,\delta _n=1$, $\alpha _1\,\delta _n=10$.

This behaviour of $\phi _{KL}(C_i)$ for small $\delta _n$ sheds light on the fact that $\alpha$ is not estimable in this model. Indeed, consider a sequence of embedded $n_k$-point designs ${\textbf{X}}_{n_k}$, initialised with the design ${\textbf{X}}_n={\textbf{X}}_{n_0}$ considered above and with $n_k=2^k\,(n_0-1)+1$, all these designs having the form $x_i=(i-1)/(n_k-1)$, $i=1,\ldots ,n_k$. Then, $\textsf{CR}({\textbf{X}}_{n_k})=\textsf{CR}({\textbf{X}}_j)=\delta _j=1/[2(n_k-1)]$ for $j=n_k,\ldots ,n_{k+1}-1=2\,n_k-2$. For *k* large enough, the increase in Kullback–Leibler divergence (10) from ${\textbf{X}}_{n_k}$ to ${\textbf{X}}_{n_{k+1}}$ is thus bounded by $c/(n_k-1)^2$ for some $c>0$, so that the expected log-likelihood ratio ${\textsf{E}}_0\{L_{n_k}\}-{\textsf{E}}_1\{L_{n_k}\}$ remains bounded as $k\rightarrow \infty$.

More generally, denote by $0\le x_1\le x_2 \le \cdots \le x_n\le 1$ the ordered points of an *n*-point design ${\textbf{X}}_n$ in [0, 1], $n\ge 3$. Let $i^*\ge 3$ be such that $|x_{i^*-2}-x_{i^*}|=\min _{i=3,\ldots ,n} |x_{i-2}-x_i |$. Then necessarily $|x_{i^*-2}-x_{i^*}|\le 1/(\lceil n/2 \rceil -1)$. Indeed, consider the following iterative modification of ${\textbf{X}}_n$ that cannot decrease $\min _{i=3,\ldots ,n} |x_{i-2}-x_i |$: first, move $x_1$ to zero, then move $x_2$ to $x_1$; leave $x_3$ unchanged, but move $x_4$ to $x_3$, etc. For *n* even, the design ${\textbf{X}}_n'$ obtained is the duplication of an (*n*/2)-points design; for *n* odd, only the right-most point $x_n$ remains single. In the fist case, the minimum distance between points of ${\textbf{X}}_n'$ is at most $1/(n/2-1)$, in the second case it is at most $1/(\lceil n/2 \rceil -1)$. We then define ${\textbf{X}}_{n-1}={\textbf{X}}_n\setminus \{x_{i^*-1}\}$. For *n* large enough, the increase in Kullback–Leibler divergence (10) from ${\textbf{X}}_{n-1}$ to ${\textbf{X}}_n$ is thus bounded by $c/(\lceil n/2 \rceil -1)^3$ for some $c>0$ depending on $\alpha _0$ and $\alpha _1$. Starting from some design ${\textbf{X}}_{n_0}$, we thus have, for $n_0$ large enough,

$$\begin{aligned} \Phi _{KL\,[K_{0},K_{1}]}({\textbf{X}}_n)-\Phi _{KL\,[K_{0},K_{1}]}({\textbf{X}}_{n_0}) \le c \sum _{k=n_0+1}^n \frac{1}{(\lceil k/2 \rceil -1)^3} , \end{aligned}$$

which implies $\lim _{n\rightarrow \infty } \Phi _{KL\,[K_{0},K_{1}]}({\textbf{X}}_n) \le B$ for some $B<\infty$. Assuming, without any loss of generality, that model 0 is correct, we have $0 \le {\textsf{E}}_0\{L_n\} \le B$ (we get $0 \le {\textsf{E}}_1\{-L_n\} \le B$ when we assume that model 1 is correct), implying in particular that $L_n$ does not tend to infinity a.s. and the ML estimator of $\alpha$ is not strongly consistent.

**Fig. 1:**
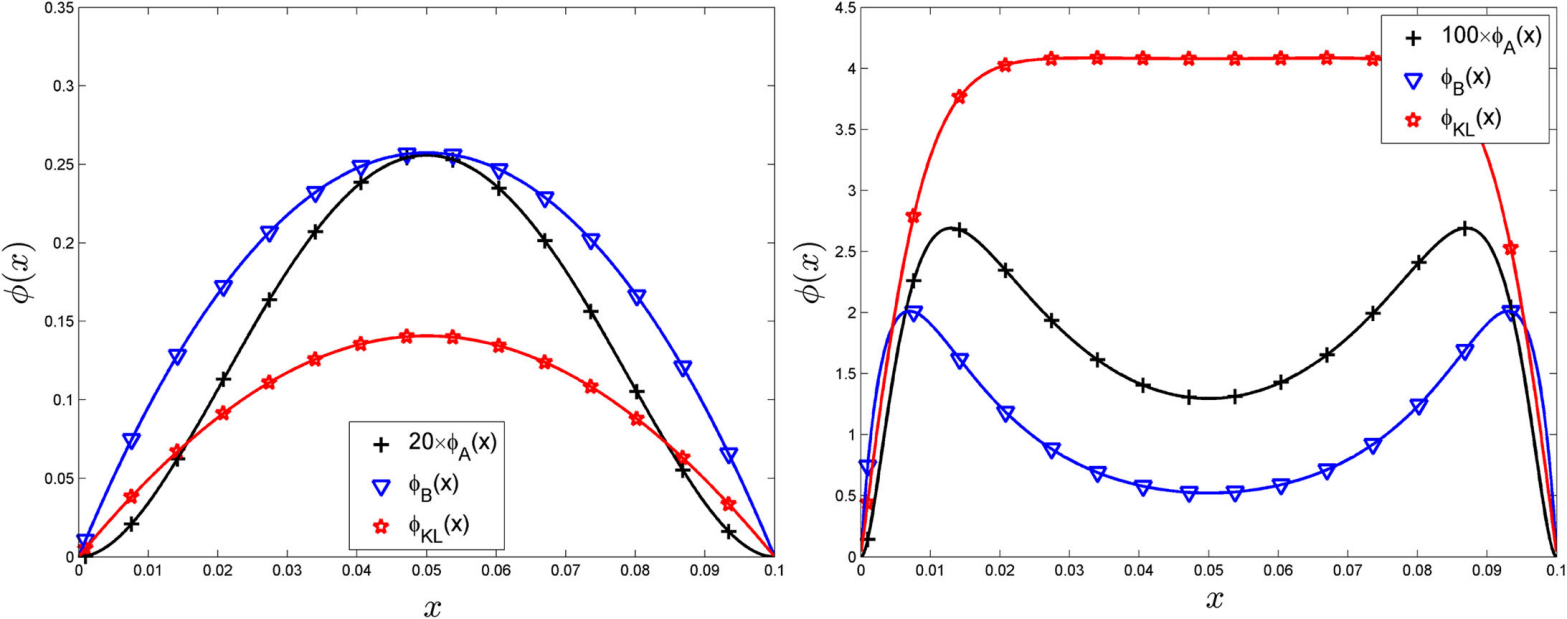
$\phi _A(x)$, $\phi _B(x)$ and $\phi _{KL}(x)$, $x\in [x_1,x_2]$, for $n=11$ ($\delta _n= 0.05$) in Example 1. Left: $\alpha _0=1$, $\alpha _1=10$; Right: $\alpha _0=20$, $\alpha _1=200$

#### Example 2: exponential covariance, microergodic parameters

Consider now two exponential covariance models with identical variances (which we take equal to one without any loss of generality): $K_i(x,x')={\textsf{e}}^{-\alpha _i|x-x'|}$, $i=0,1$.

Again, $\phi _A(x) \rightarrow 0$ as $x\rightarrow x_i\in {\textbf{X}}_n$ and $\phi _A(\cdot )$ has a unique maximum at $C_i$ for small enough $\delta _n$, with now

$$\begin{aligned} \phi _A(C_i)= \frac{1}{2}\, \left( \alpha _1^2-\alpha _0^2\right) ^2 \, \delta _n^4+ {\mathcal {O}}\left( \delta _n^5\right) , \ n\rightarrow \infty . \end{aligned}$$

There are two maxima for $\phi _A(\cdot )$ in ${\mathbb {I}}_i$, symmetric with respect to $C_i$ for large $\delta _n$: when $\alpha _0\,\delta _n=1$, this happens for instance when $\alpha _1\,\delta _n \gtrsim 2.558545$. Nothing is changed for $\phi _B(\cdot )$ compared to Example 1 as the variances cancel in the ratios that define $\phi _B(\cdot )$, see (3) and (6). The situation is quite different for $\phi _{KL}(\cdot )$, with

$$\begin{aligned} \phi _{KL}(C_i)= \frac{1}{2}\, \frac{(\alpha _1-\alpha _0)^2}{\alpha _0\alpha _1} + {\mathcal {O}}(\delta _n) , \ n\rightarrow \infty , \end{aligned}$$

indicating that it is indeed possible to distinguish between the two models much more efficiently with this criterion than with the two others. Interestingly enough, the best choice for next design point is not at $C_i$ but always as close as possible to one of the endpoints $a_i$ or $b_i$, with however a criterion value similar to that in the centre $C_i$ when $\delta _n$ is small enough, as $\lim _{x\rightarrow x_i} \phi _{KL}(x) = (\alpha _1-\alpha _0)^2/(2\,\alpha _0\alpha _1)$. Here, the same sequence of embedded designs as in Example 1 ensures that ${\textsf{E}}_0\{L_{n_k}\}-{\textsf{E}}_1\{L_{n_k}\} \rightarrow \infty$ as $k\rightarrow \infty$. Figure  presents $\phi _A(x)$, $\phi _B(x)$ and $\phi _{KL}(x)$ in the same configuration as in Fig. 1 but for the kernels $K_i(x,x')={\textsf{e}}^{-\alpha _i|x-x'|}$, $i=0,1$.

**Fig. 2:**
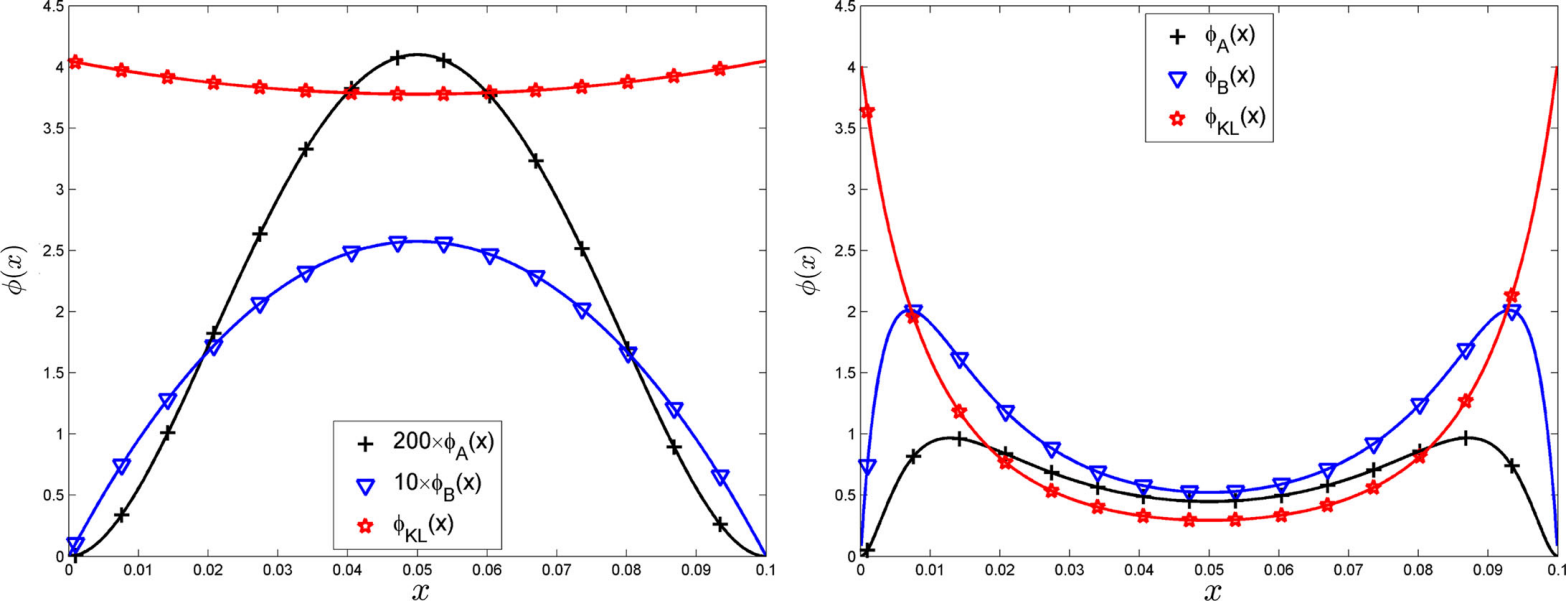
$\phi _A(x)$, $\phi _B(x)$ and $\phi _{KL}(x)$, $x\in [x_1,x_2]$, for $n=11$ ($\delta _n= 0.05$) in Example 2. Left: $\alpha _0=1$, $\alpha _1=10$; Right: $\alpha _0=20$, $\alpha _1=200$

#### Example 3: Matérn kernels

Take $K_0$ and $K_1$ as the 3/2 and 5/2 Matérn kernels, respectively:

8 $$\begin{aligned} K_{0,\theta }(x,x')= & {} (1+\sqrt{3}\theta \,|x-x' |)\, \exp (-\sqrt{3}\theta \,|x-x'|) \ \text{(Mat }\acute{{\textrm{e}}}\text{ rn } \text{3/2) }, \end{aligned}$$

9 $$\begin{aligned} K_{1,\theta }(x,x')= & {} [1+\sqrt{5}\theta \, |x-x'|+ 5\theta ^2\, |x-x'|^2/3]\,\exp (-\sqrt{5}\theta \, |x-x'|) \ \text{(Mat }\acute{{\textrm{e}}}\text{ rn } \text{5/2) }. \nonumber \\ \end{aligned}$$

We take $\theta =\theta _0=1$ in $K_{0,\theta }$ and adjust $\theta =\theta _1$ in $K_{1,\theta }$ to minimise $\phi _{2\,[K_{0,\theta _0},K_{1,\theta _1}]}(\mu )$ defined by Eq. (13) in Sect. 4 with $\mu$ the uniform measure on [0, 1], which gives $\theta _1\simeq 1.1275$. The left panel of Fig.  shows $K_{0,\theta _0=1}(x,0)$ and $K_{1,\theta }(x,0)$ for $\theta =1$ and $\theta =\theta _1$ when $x\in [0,1]$. The right panel presents $\phi _B(x)$ and $\phi _{KL}(x)$ for the same $n=11$-point equally spaced design ${\textbf{X}}_n$ as in Example 1 and $x\in [0,1]$ for $K_{0,1}$ and $K_{1,1.1275}$ (the value of $\phi _A(x)$ does not exceed $0.65\,10^{-4}$ and is not shown). The behaviours of $\phi _B(x)$ and $\phi _{KL}(x)$ are now different in different intervals $[x_i,x_{i+1}]$ (they remain symmetric with respect to 1/2, however), the maximum of $\phi _{KL}(x)$ is obtained at the central point $x_5$. The behaviour of $\phi _{KL}(\cdot )$ could be related to the fact that discriminating between $K_0$ and $K_1$ amounts to estimating the smoothness of the realisation, which requires that some design points are close to each other.

**Fig. 3:**
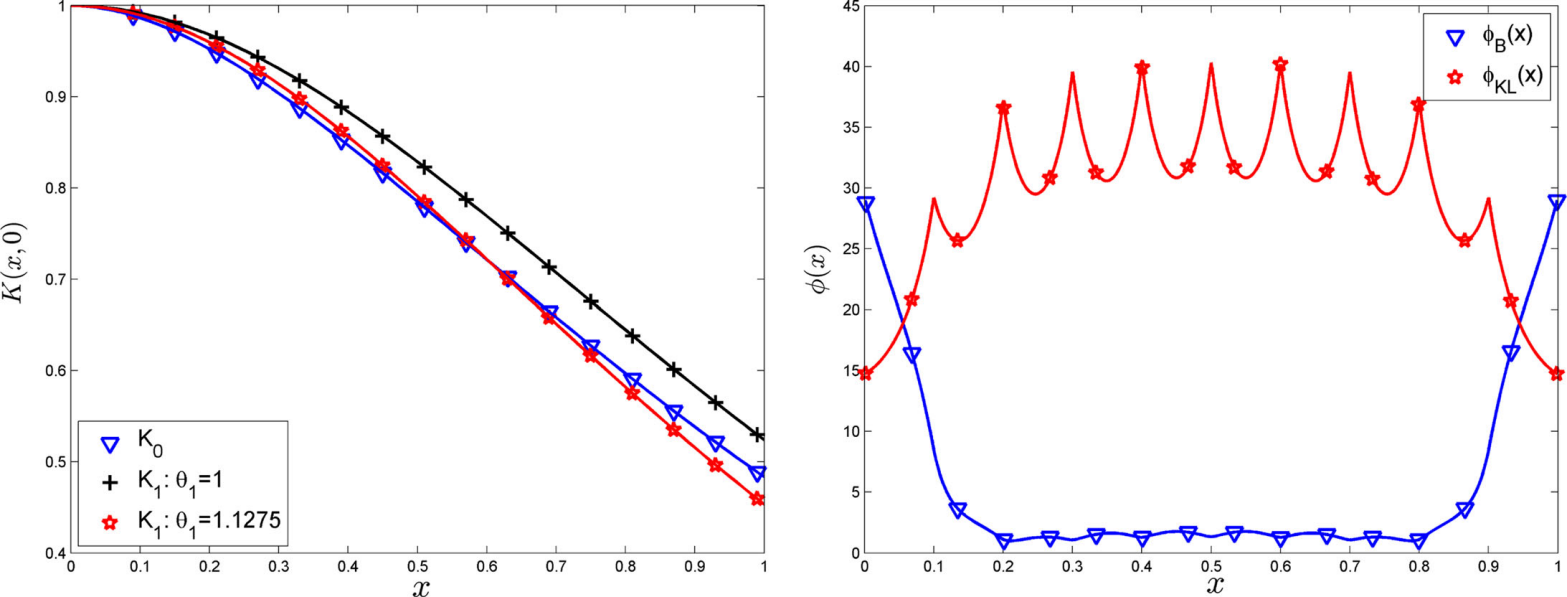
Left: $K_{0,1}(x,0)$, $K_{1,1}(x,0)$ and $K_{1,1.1275}(x,0)$, $x\in [0,1]$. Right: $\phi _B(x)$ and $\phi _{KL}(x)$ for $x\in [0,1]$ and the same 11-point equally spaced design ${\textbf{X}}_n=\{0,1/10,2/10,\ldots ,1\}$ as in Example 1, with $K_{0,1}$ and $K_{1,1.1275}$

## Distance-based discrimination

We will now consider criteria which are directly based on the discrepancies of the covariance kernels. Ideally those should be simpler to compute and still exhibit reasonable efficiencies and some similar properties. The starting point is again the use of the log-likelihood ratio criterion to choose among the two models. Assuming that the random field is Gaussian, the probability densities of observations ${\textbf{Y}}_n$ for the two models are

$$\begin{aligned} \varphi _{n,i}({\textbf{Y}}_n) = \frac{1}{(2\pi )^{n/2}\,\det ^{1/2} {\textbf{K}}_{n,i}} \, \exp \left[ - \frac{1}{2}\, {\textbf{Y}}_n^\top {\textbf{K}}_{n,i}^{-1} {\textbf{Y}}_n \right] , \ i=0,1. \end{aligned}$$

The expected value of the log-likelihood ratio $L_n=\log \varphi ({\textbf{Y}}_n \arrowvert 0)- \log \varphi ({\textbf{Y}}_n \arrowvert 1)$ under model 0 is

$$\begin{aligned} {\textsf{E}}_0\{L_n\} = \frac{1}{2} \log \det \left( {\textbf{K}}_{n,1}{\textbf{K}}_{n,0}^{-1}\right) - \frac{n}{2} +\frac{1}{2}\, \textrm{trace}\left( {\textbf{K}}_{n,0}{\textbf{K}}_{n,1}^{-1}\right) \end{aligned}$$

and similarly

$$\begin{aligned} {\textsf{E}}_1\{L_n\} = \frac{1}{2} \log \det \left( {\textbf{K}}_{n,1}{\textbf{K}}_{n,0}^{-1}\right) + \frac{n}{2} - \frac{1}{2}\, \textrm{trace}\left( {\textbf{K}}_{n,1}{\textbf{K}}_{n,0}^{-1}\right) . \end{aligned}$$

A good discriminating design should make the difference ${\textsf{E}}_0\{L_n\}-{\textsf{E}}_1\{L_n\}$ as large as possible; that is, we should choose ${\textbf{X}}_n$ that maximises

10 $$\begin{aligned}{} & {} \Phi _{KL\,[K_0,K_1]}({\textbf{X}}_n) \nonumber \\ {}{} & {} \quad = {\textsf{E}}_0\{L_n\}-{\textsf{E}}_1\{L_n\}= \frac{1}{2} \left[ \textrm{trace}\left( {\textbf{K}}_{n,0}{\textbf{K}}_{n,1}^{-1}\right) + \textrm{trace}\left( {\textbf{K}}_{n,1}{\textbf{K}}_{n,0}^{-1}\right) \right] - n \, \nonumber \\{} & {} = 2\, D_{KL}\left( \varphi _{n,0},\varphi _{n,1}\right) , \end{aligned}$$

i.e. twice the symmetric Kullback–Leibler divergence between the normal distributions with densities $\varphi _{n,0}$ and $\varphi _{n,1}$, see, e.g., Pronzato et al. (2019).

We may enforce the normalisation $\sigma _0^2=\sigma _1^2=1$ and choose the $\theta _i$ to make the two kernels most similar in the sense of the criterion $\Phi$ considered; that is, maximise

11 $$\begin{aligned} \min _{\theta _0\in \Theta _0,\,\theta _1\in \Theta _1}\Phi _{KL\,[K_0,K_1]}({\textbf{X}}_n). \end{aligned}$$

The choice of $\Theta _0$ and $\Theta _1$ is important; in particular, unconstrained minimisation over the $\theta _i$ could make both kernels completely flat or on the opposite close to Dirac distributions. It may thus be preferable to fix $\theta _0$ and minimise over $\theta _1$ without constraints. Also, the Kullback–Leibler distance is sensitive to kernel matrices being near singularity, which might happen if design points are very close to each other. Pronzato et al. (2019) suggest a family of criteria based on matrix distances derived from Bregman divergences between functions of covariance matrices from Kiefer’s $\varphi _p$-class of functions (Kiefer 1974). If $p \in (0,1)$, these criteria are rather insensitive to eigenvalues close or equal to zero. Alternatively, they suggest criteria computed as Bregman divergences between squared volumes of random *k*-dimensional simplices for $k \in \{2,\ldots ,d-1\}$, which have similar properties.

The index *n* is omitted in the following and we consider fixed parameters for both kernels. The Fréchet-distance criterion

12 $$\begin{aligned} \Phi _{F\,[K_0,K_1]}({\textbf{X}}_n) = \textrm{trace}\left[ {\textbf{K}}_0+{\textbf{K}}_1-2\,({\textbf{K}}_0{\textbf{K}}_1)^{1/2}\right] , \end{aligned}$$

related to the Kantorovich (Wasserstein) distance, seems of particular interest due to the absence of matrix inversion. The expression is puzzling since the two matrices do not necessarily commute, but the paper Dowson and Landau (1982) is illuminating.

Other matrix “entry-wise" distances will be considered, in particular the one based on the (squared) Frobenius norm,

$$\begin{aligned} \Phi _{2\,[K_0,K_1]}({\textbf{X}}_n) = \textrm{trace}\left( {\textbf{K}}_0^2+{\textbf{K}}_1^2-2\,{\textbf{K}}_0{\textbf{K}}_1\right) = \textrm{trace}\left[ ({\textbf{K}}_0-{\textbf{K}}_1)^2\right] , \end{aligned}$$

which corresponds to the substitution of ${\textbf{K}}_i^2$ for ${\textbf{K}}_i$ in (12) for $i=0,1$. Denote more generally

$$\begin{aligned} \Phi _{p\,[K_0,K_1]}({\textbf{X}}_n) = \Vert {\textbf{K}}_1-{\textbf{K}}_0\Vert _p^p = \sum _{i,j=1}^n |\{{\textbf{K}}_1-{\textbf{K}}_0\}_{i,j}|^p = {\textbf{1}}_n^\top |{\textbf{K}}_1-{\textbf{K}}_0|^{\odot p} {\textbf{1}}_n , \ p>0 , \end{aligned}$$

where ${\textbf{1}}_n$ is the *n*-dimensional vector with all components equal to 1, the absolute value is applied entry-wise and $^{\odot p}$ denotes power *p* applied entry-wise.

Figure  shows the values of the criteria $\Phi _{i\,[K_{0,1},K_{1,\theta }]}$, $i=1,2$, $\Phi _{F\,[K_{0,1},K_{1,\theta }]}$ and $\Phi _{KL\,[K_{0,1},K_{1,\theta }]}$ as functions of $\theta$ for the two kernels $K_{0,\theta }$ and $K_{1,\theta }$ given by (8) and (9) and the same regular design as in Example 1: $x_i=(i-1)/(n-1)$, $i=1,\ldots ,11$. The criteria are re-scaled so that their maximum equals one on the interval considered for $\theta$. Note the similarity between $\Phi _{2\,[K_{0,1},K_{1,\theta }]}({\textbf{X}}_n)$ and $\Phi _{F\,[K_{0,1},K_{1,\theta }]}({\textbf{X}}_n)$ and the closeness between the distance-minimising $\theta$ for $\Phi _1$, $\Phi _2$ and $\Phi _F$. Also note the good agreement with the value $\theta _1\simeq 1.1275$ that minimises $\phi _{2\,[K_{0,1},K_{1,\theta _1}]}(\mu )$ from Eq. (13), see Example 3. The optimal $\theta$ for $\Phi _{KL\,[K_{0,1},K_{1,\theta }]}({\textbf{X}}_n)$ is much different, however, showing that the criteria do not necessarily agree between them.

**Fig. 4:**
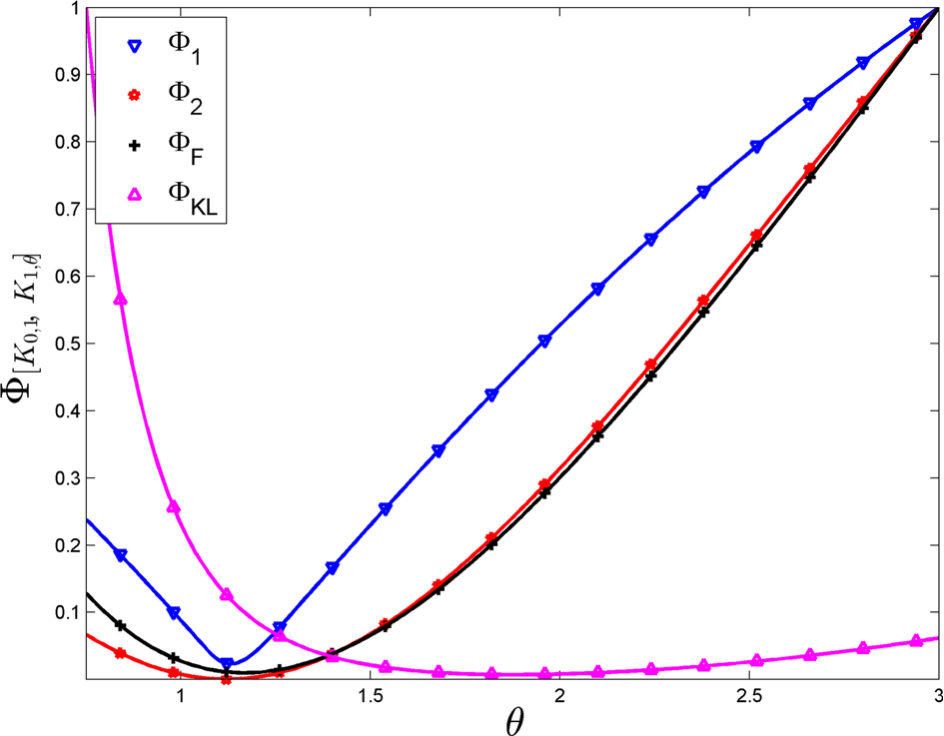
$\Phi _{i\,[K_{0,1},K_{1,\theta }]}({\textbf{X}}_n)$, $i=1,2$, $\Phi _{F\,[K_{0,1},K_{1,\theta }]}({\textbf{X}}_n)$ and $\Phi _{KL\,[K_{0,1},K_{1,\theta }]}({\textbf{X}}_n)$ as functions of $\theta \in [0.75,3]$ for the same 11-point equally spaced design ${\textbf{X}}_n$ as in Example 1 and $K_{0,\theta }$, $K_{1,\theta }$ given by (8) and (9), respectively

An interesting feature of the family of criteria $\Phi _{p\,[K_0,K_1]}(\cdot )$, $p>0$, is that they extend straightforwardly to a design measure version. Indeed, defining $\xi _n$ as the empirical measure on the points in ${\textbf{X}}_n$, $\xi _n=(1/n)\,\sum _{i=1}^n \delta _{x_i}$, we can write

$$\begin{aligned} \Phi _{p\,[K_0,K_1]}({\textbf{X}}_n) = n^2\, \phi _{p\,[K_0,K_1]}(\xi _n) , \end{aligned}$$

where we define, for any design (probability) measure on ${{\mathscr {X}}}$,

13 $$\begin{aligned} \phi _p(\xi ) = \phi _{p\,[K_0,K_1]}(\xi )= & {} \int _{{{\mathscr {X}}}^2} |K_1(x,x')-K_0(x,x')|^p \, \textrm{d}\xi (x)\textrm{d}\xi (x') . \end{aligned}$$

Denote by $F_{p\,[K_0,K_1]}(\xi ;\nu )$ the directional derivative of $\phi _{p\,[K_0,K_1]}(\cdot )$ at $\xi$ in the direction $\nu$,

$$\begin{aligned} F_{p\,[K_0,K_1]}(\xi ;\nu ) = \lim _{\alpha \rightarrow 0^+} \frac{\phi _{p\,[K_0,K_1]}[(1-\alpha )\xi +\alpha \nu ]-\phi _{p\,[K_0,K_1]}(\xi )}{\alpha } . \end{aligned}$$

Direct calculation gives

$$\begin{aligned} F_{p\,[K_0,K_1]}(\xi ;\nu ) = 2 \left[ \int _{{{\mathscr {X}}}^2} |K_1(x,x')-K_0(x,x')|^p\, \textrm{d}\nu (x)\textrm{d}\xi (x') - \phi _{p\,[K_0,K_1]}(\xi ) \right] , \end{aligned}$$

and thus in particular

$$\begin{aligned} F_{p\,[K_0,K_1]}(\xi ;\delta _x) = 2 \left[ \int _{{\mathscr {X}}}|K_1(x,x')-K_0(x,x')|^p\, \textrm{d}\xi (x') - \phi _{p\,[K_0,K_1]}(\xi ) \right] . \end{aligned}$$

One can easily check that the criterion is neither concave nor convex in general (as the matrix $|{\textbf{K}}_1-{\textbf{K}}_0|^{\odot p}$ can have both positive and negative eigenvalues), but we nevertheless have a necessary condition for optimality.

### Theorem 1

If the probability measure $\xi ^*$ on ${{\mathscr {X}}}$ maximises $\phi _{p\,[K_0,K_1]}(\xi )$, then

14 $$\begin{aligned} \forall x\in {{\mathscr {X}}}, \ \int _{{\mathscr {X}}}|K_1(x,x')-K_0(x,x')|^p\, \textrm{d}\xi ^*(x') \le \phi _{p\,[K_0,K_1]}(\xi ^*) . \end{aligned}$$

Moreover, $\int _{{\mathscr {X}}}|K_1(x,x')-K_0(x,x')|^p\, \textrm{d}\xi ^*(x')=\phi _{p\,[K_0,K_1]}(\xi ^*)$ for $\xi ^*$-almost every $x\in {{\mathscr {X}}}$.

The proof follows from the fact that $F_{p\,[K_0,K_1]}(\xi ^*;\nu )\le 0$ for every $\nu$ when $\xi ^*$ is optimal, which implies (14). As $\int _{{\mathscr {X}}}\left[ \int _{{\mathscr {X}}}|K_1(x,x')-K_0(x,x')|^p\, \textrm{d}\xi ^*(x')\right] \textrm{d}\xi ^*(x)=\phi _{p\,[K_0,K_1]}(\xi ^*)$, the inequality necessarily becomes an equality on the support of $\xi ^*$.

This suggests the following simple incremental construction: at iteration *n*, with ${\textbf{X}}_n$ the current design and $\xi _n$ the associated empirical measure, choose $x_{n+1}\in \textrm{Arg}\max _{x\in {{\mathscr {X}}}} F_{p\,[K_0,K_1]}(\xi _n;\delta _x)= \textrm{Arg}\max _{x\in {{\mathscr {X}}}} {\textbf{1}}_n^\top |{\textbf{k}}_{n,0}(x)-{\textbf{k}}_{n,1}(x)|^{\odot p}$. It will be used in the numerical example of Sect. 6.2.

## Optimal design measures

In this section we explain why the determination of optimal design measures maximising $\phi _p(\xi )$ is generally difficult, even when limiting ourselves to the satisfaction of the necessary condition in Theorem 1. At the same time, we can characterise measures that are approximately optimal for large *p*.

We assume that the two kernels are isotropic, i.e., such that $K_i(x,x')=\Psi _i(\Vert x-x'\Vert )$, $i=0,1$, and that the functions $\Psi _i$ are differentiable except possibly at 0 where they only admit a right derivative. We define $\psi (t)=|\Psi _1(t)-\Psi _0(t)|$, $t\in {\mathbb {R}}^+$, and assume that the kernels have been normalised so that $K_0(x,x)=K_1(x,x)$; that is, $\psi (0)=0$. Also, we only consider the case where the function $\psi (\cdot )$ has a unique global maximum on ${\mathbb {R}}^+$. This assumption is not very restrictive. Consider again the two Matérn kernels (8) and (9). Figure  shows the evolution of $\psi ^2(t)$ for $K_0=K_{0,1}$ and $K_1=K_{1,\theta _1}$ with two different values of $\theta _1$: $\theta _1=1$ and $\theta _1\simeq 1.1275$; the latter minimises $\phi _{2\,[K_{0,1},K_{1,\theta }]}(\mu )$ for $\mu$ being the uniform measure on [0, 1].

**Fig. 5:**
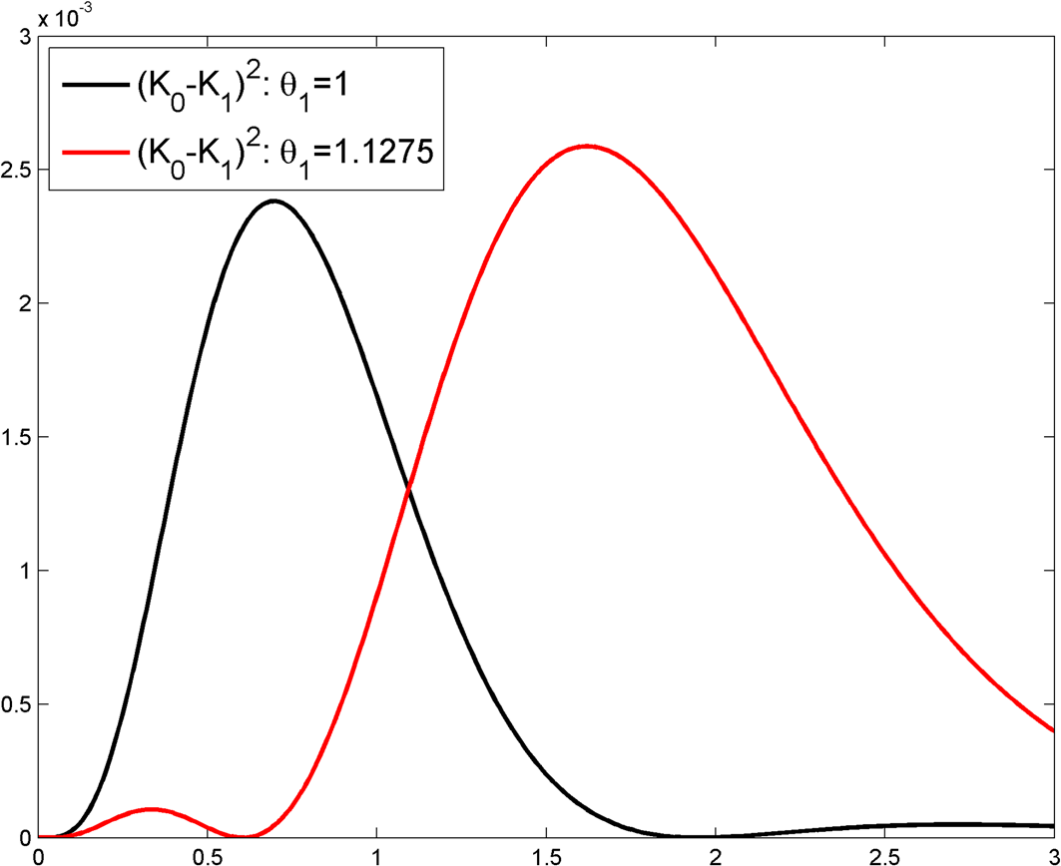
$\psi ^2(t)$ for $K_0=K_{0,1}$ and $K_1=K_{1,\theta _1}$ with two different values of $\theta _1$

In the following, we shall consider normalised functions $\psi (\cdot )$, such that $\max _{t\in {\mathbb {R}}^+} \psi (t)=1$. We denote by $\Delta$ the (unique) value such that $\psi (\Delta )=1$. On Fig. 5, $\Delta \simeq 0.7$ when $K_1=K_{1,1}$.

### A simplified problem with an explicit optimal solution

Consider the extreme case where $\psi =\psi _*$ defined by

15 $$\begin{aligned} \psi _*(t) = \left\{ \begin{array}{ll} 1 &{}\quad \text{ if } t=\Delta , \\ 0 &{}\quad \text{ otherwise. } \end{array}\right. \end{aligned}$$

Note that $\psi _*^p(t)=\psi _*(t)$ for any $p>0$; we can thus restrict our attention to $p=1$ for the maximisation of $\phi _p(\xi )$ defined by (13); that is, we consider

$$\begin{aligned} \phi _1(\xi ) = \int _{{{\mathscr {X}}}^2} \psi _*(\Vert x-x'\Vert ) \, \textrm{d}\xi (x)\textrm{d}\xi (x'). \end{aligned}$$

#### Theorem 2

When $\psi =\psi _*$ and ${{\mathscr {X}}}\subset {\mathbb {R}}^d$ is large enough to contain a regular *d* simplex with edge length $\Delta$, any measure $\xi ^*$ allocating weight $1/(d+1)$ at each vertex of such a simplex maximises $\phi _1(\xi )$, and $\phi _1(\xi ^*)=d/(d+1)$.

#### Proof

Since $\phi _1(\xi )=0$ when $\xi$ is continuous with respect to the Lebesgue measure on ${{\mathscr {X}}}$, we can restrict our attention to measures without any continuous component. Assume that $\xi =\sum _{i=1}^n w_i \delta _{x_i}$, with $w_i\ge 0$ for all *i* and $\sum _{i=1}^n w_i=1$, $n\in {\mathbb {N}}$. Consider the graph ${\mathcal {G}}(\xi )$ having the $x_i$ as vertices, with an edge (*i*, *j*) connecting $x_i$ and $x_j$ if and only if $\Vert x_i-x_j\Vert = \Delta$. We have

$$\begin{aligned} \phi _1(\xi ) = \sum _{(i,j)\in {\mathcal {G}}(\xi )} w_i w_j, \end{aligned}$$

and Theorem 1 of Motzkin and Straus (1965) implies that $\phi _1(\xi )$ is maximum when $\xi$ is uniform on the maximal complete subgraph of ${\mathcal {G}}(\xi )$. The maximal achievable order is $d+1$, obtained when the $x_i$ are the vertices of a regular simplex in ${{\mathscr {X}}}$ with edge length $\Delta$. Motzkin and Straus (1965) also indicate in their Theorem 1 that $\phi _1(\xi ^*)=1-1/(d+1)$. This is easily recovered knowing that ${\mathcal {G}}(\xi ^*)$ is fully connected with order $d+1$. Indeed, we then have

$$\begin{aligned} \phi _1(\xi )=\sum _{i=1}^{d+1} w_i \sum _{{\mathop {j\ne i}\limits ^{j=1}}}^{d+1} w_j = \sum _{{\mathop {j\ne i}\limits ^{i,j=1}}}^{d+1} w_iw_j = 1-\sum _{i=1}^{d+1} w_i^2, \end{aligned}$$

which is maximum when all $w_i$ equal $1/(d+1)$. $\square$

### Optimal designs for $\psi (t)=|\Psi _1(t)-\Psi _0(t)|$

The optimal designs of Theorem 2 are natural candidates for being optimal when we return to the case of interest $\psi (t)=|\Psi _1(t)-\Psi _0(t)|$. In the light of Theorem 1, for a given probability measure $\xi$ on ${{\mathscr {X}}}$, we consider the function

$$\begin{aligned} \delta _\xi (x)=\int _{{\mathscr {X}}}\psi ^p(\Vert x-x'\Vert )\, \textrm{d}\xi (x') - \phi _p(\xi ), \end{aligned}$$

which must satisfy $\delta _\xi (x)\le 0$ for all $x\in {{\mathscr {X}}}$ when $\xi$ is optimal. For an optimal measure $\xi ^*$ as in Theorem 2, with support $x_1,\ldots ,x_{d+1}$ forming a regular *d*-simplex, we have

$$\begin{aligned} \delta _{\xi ^*}(x)= \frac{1}{d+1} \, \left[ \sum _{i=1}^{d+1} \psi ^p(\Vert x-x_i\Vert ) - d \right] . \end{aligned}$$

One can readily check that $\delta _{\xi ^*}(x_i)=0$ for all *i* (as $\psi (\Vert x_i-x_j\Vert )=\psi (\Delta )=1$ for $i\ne j$ and $\psi (0)=0$). Moreover, since $\psi (\cdot )$ is differentiable everywhere except possibly at zero, when $p>1$ the gradient of $\delta _{\xi ^*}(x)$ equals zero at each $x_i$. However, these $d+1$ stationary points may sometimes correspond to local minima—a situation when of course $\xi ^*$ is not optimal. The left panel of Fig.  shows an illustration ($d=2$) for $p=1.5$, $K_0(x,x')=\exp (-\Vert x-x'\Vert )$ and $K_1$ being the Matérn 5/2 kernel $K_{1,1}$. The measure $\xi ^*$ is supported at the vertices of the equilateral triangle $(0,0),(\Delta ,0),(\Delta /2,\sqrt{(}3)\Delta /2)$ (indicated in blue on the figure), with $\Delta \simeq 0.53$ (the value where $\psi (\cdot )$ is maximum). Here the $x_i$ correspond to local minima of $\delta _{\xi ^*}(x)$, $\psi (\cdot )$ is not differentiable at zero but $p>1$ so that $\delta _{\xi ^*}(\cdot )$ is differentiable.

When $p\rightarrow \infty$, $\psi ^p(\cdot )$ approaches the (discontinuous) function $\psi _*(\cdot )$, suggesting that $\xi ^*$ may become close to being optimal for $\phi _p$ when *p* is large enough. However, when ${{\mathscr {X}}}$ is large, $\xi ^*$ is never truly optimal, no matter how large *p* is. Indeed, suppose that ${{\mathscr {X}}}$ contains a point $x_*$ corresponding to the symmetric of a vertex $x_k$ of the simplex defining the support of $\xi ^*$ with respect to the opposite face of that simplex. Direct calculation gives

$$\begin{aligned} L=\Vert x_k-x_*\Vert =2\,\Delta \,\left( \frac{d+1}{2\,d}\right) ^{1/2}. \end{aligned}$$

**Fig. 6:**
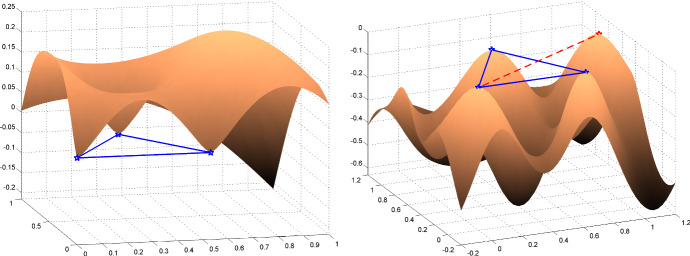
Surface plot of $\delta _{\xi ^*}(x)$ ($x\in {\mathbb {R}}^2$), the support of $\xi ^*$ corresponds to the vertices of the equilateral triangle in blue. Left: $K_0(x,x')=\exp (-\Vert x-x'\Vert )$ and $K_1=K_{1,1}$ ($\Delta \simeq 0.53$), $p=1.5$; Right: $K_0=K_{0,1}$, $K_1=K_{1,1}$ ($\Delta \simeq 0.7$), $p=10$; the red point $x_*$ is the symmetric of the origin (0, 0) with respect to the opposite side of the triangle. (Color figure online)

The right panel of Fig. 6 shows an illustration for $K_0$ and $K_1$ being the Matérn 3/2 and Matérn 5/2 kernels $K_{0,1}$ and $K_{1,1}$, respectively. The measure $\xi ^*$ is supported at the vertices of the equilateral triangle with vertices $(0,0),(\Delta ,0),(\Delta /2,\sqrt{(}3)\Delta /2)$ with now $\Delta \simeq 0.7$. At the point $x_*$, symmetric to $x_k$, indicated in red on the figure, we have

16 $$\begin{aligned} \delta _{\xi ^*}(x_*)= & {} \frac{1}{d+1} \, \left[ \sum _{{\mathop {i\ne k}\limits ^{i=1}}}^{d+1} \psi ^p(\Vert x_*-x_i\Vert ) + \psi ^p(\Vert x_*-x_k\Vert ) - d \right] \nonumber \\= & {} \frac{1}{d+1}\, \psi ^p(L) >0 , \end{aligned}$$

where the second equality follows from $\Vert x_*-x_i\Vert =\Delta$ for all $i\ne k$, implying that $\xi ^*$ is not optimal. Another, more direct, proof of the non-optimality of $\xi ^*$ is to consider the measure ${{\widehat{\xi }}}$ that sets weights $1/(d+1)$ at all $x_i\ne x_k$ and weights $1/[2(d+1)]$ at $x_k$ and its symmetric $x_*$. Direct calculation gives

$$\begin{aligned} \phi _p({{\widehat{\xi }}}) = \frac{d}{d+1}\,\left( 1-\frac{1}{d+1}\right) + \frac{2}{2\,(d+1)}\left[ \frac{d}{d+1}+\frac{1}{2\,(d+1)} \,\psi ^p(L)\right] . \end{aligned}$$

The first term on the right-hand side comes from the *d* vertices $x_i$, $i\ne k$, each one having weight $1/(d+1)$ and being at distance $\Delta$ of all other vertices, those having total weight $1-1/(d+1)$. The second term comes from the two symmetric points $x_k$ and $x_*$, each one with weight $1/[2(d+1)]$. Each of these two points is at distance $\Delta$ from *d* vertices with weights $1/(d+1)$ and at distance *L* of the other opposite point with weight $1/[2(d+1)]$. We get after simplification

$$\begin{aligned} \phi _p({{\widehat{\xi }}}) = \frac{d}{d+1} + \frac{\psi ^p(L)}{2\,(d+1)^2} > \phi _p(\xi ^*) = \frac{d}{d+1}, \end{aligned}$$

showing that $\xi ^*$ is not optimal. Note that, for symmetry reasons, the design ${{\widehat{\xi }}}$ is not optimal for large enough ${{\mathscr {X}}}$. The determination of a truly optimal design seems very difficult. In the simplified problem of Sect. 5.1, where the criterion is based on the function $\psi _*$ defined by (15), the measures $\xi ^*$ and ${{\widehat{\xi }}}$ supported on $d+1$ and $d+2$ points, respectively, have the same criterion value $\phi _p(\xi ^*)=\phi _p({{\widehat{\xi }}})=d/(d+1)$ for all $p>0$.

Although $\xi ^*$ is not optimal, since $\psi (\Vert x_*-x_k\Vert )<1$ (as $\psi (t)$ takes its maximum value 1 for $t=\Delta$), (16) suggests that $\xi ^*$ may be only marginally suboptimal when *p* is large enough. Moreover, as the right panel of Fig. 6 illustrates, a design $\xi ^*$ supported on a regular simplex is optimal provided that ${{\mathscr {X}}}$ is small enough and *p* is large enough to make $\delta _{\xi ^*}(x)$ concave at each $x_i$ (for symmetry reasons, we only need to check concavity at one vertex). In fact, $p>2$ is sufficient. Indeed, assuming that $p>2$ and that $\psi (\cdot )$ is twice differentiable everywhere, with second-order derivative $\psi ''(\cdot )$, except possibly at zero, direct calculation gives

$$\begin{aligned} \frac{d^2\delta _{\xi ^*}(x)}{dxdx^\top } \bigg \arrowvert _{x=x_1}= \frac{1}{d+1}\, \frac{p\,\psi ^{p-1}(\Delta )\psi ''(\Delta )}{\Delta ^2} \sum _{i=2}^{d+1} (x_1-x_i)(x_1-x_i)^\top , \end{aligned}$$

which is negative-definite (since $\psi ''(\Delta )<0$, $\psi (\cdot )$ being maximal at $\Delta$). The right panel of Fig. 6 gives an illustration. Note that $p<2$ on the left panel, and the $x_i$ correspond to local minimas of $\delta _{\xi ^*}(\cdot )$. Figure  shows a plot of $\delta _{\xi ^*}(x)$ for $p=2$ and $K_0$ and $K_1$ being the Matérn 3/2 and Matérn 5/2 kernels $K_{0,1}$ and $K_{1,1.07}$, respectively, suggesting that the form of optimal designs may be in general quite complicated.

**Fig. 7:**
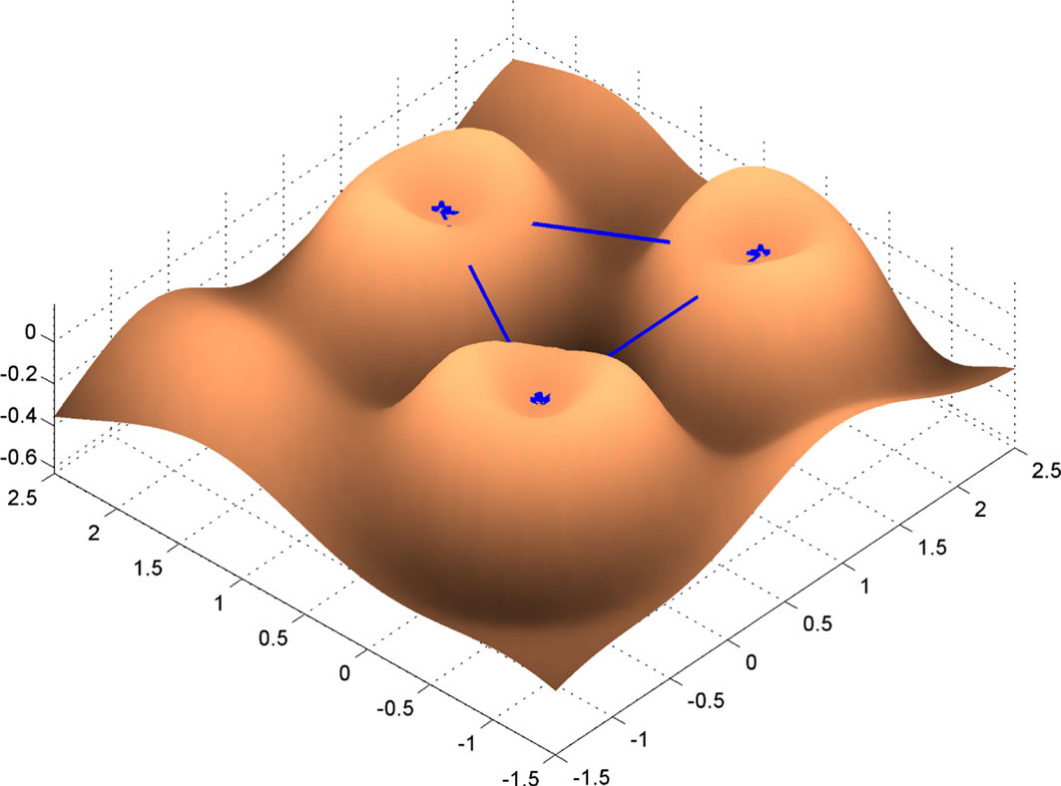
Surface plot of $\delta _{\xi ^*}(x)$ ($x\in {\mathbb {R}}^2$), the support of $\xi ^*$ corresponds to the vertices of the equilateral triangle in blue: $K_0=K_{0,1}$, $K_1=K_{1,1.07}$ ($\Delta \simeq 1.92$), $p=2$. (Color figure online)

## A numerical example

### Exact designs

In this section, we consider numerical evaluations of designs resulting from the prediction-based and distance-based criteria. Here, the rival models are the isotropic versions of the covariance kernels used in Example 3 (Sect. 3.2) for the design space ${{\mathscr {X}}}=[0,10]^2$, discretised at $n=25$ equally spaced points in each dimension. For an agreement on the setting of correlation lengths in both kernels, we have applied a minimisation procedure. Specifically, we have taken $\theta =\theta _0=1$ in $K_{0,\theta }(x,x')$ and adjusted the parameter in the second kernel minimising each of the distance-based criteria for the design ${\textbf{X}}_{625}$ corresponding to the full grid. This resulted in $\theta _1= 1.0047$, 1.0285, 1.0955 and 1.3403, respectively, for $\Phi _F,\Phi _1,\Phi _2$ and $\Phi _{KL}$. We have finally chosen $\theta _1=1.07$, which seems to be compatible with the above values.

The left panel in Fig.  shows the plot of the two Matérn covariance functions at the assumed parameter values. This plot illustrates the similarity of the kernels which we aim to discriminate. The right panel in the figure refers to the plot of the absolute difference between the covariance kernels. The red line corresponds to the distance where the absolute difference between them is maximal. This is denoted by $\Delta$, which is equal to $\Delta =1.92$ in this case.

**Fig. 8:**
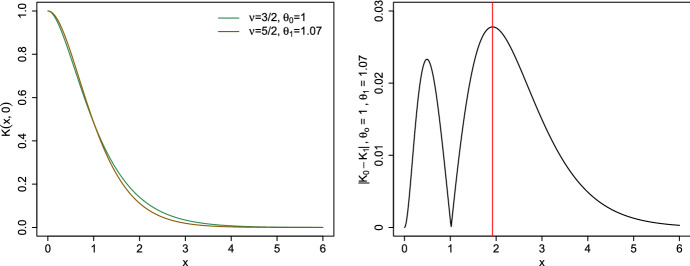
Left: Plot of the Matérn covariance functions at the assumed parameter setting. Right: $\psi (t)=|K_{0,\theta _0}(t,0)-K_{0,\theta _1}(t,0)|, (\theta _0=1,\theta _1=1.07)$. (Color figure online)

**Fig. 9:**
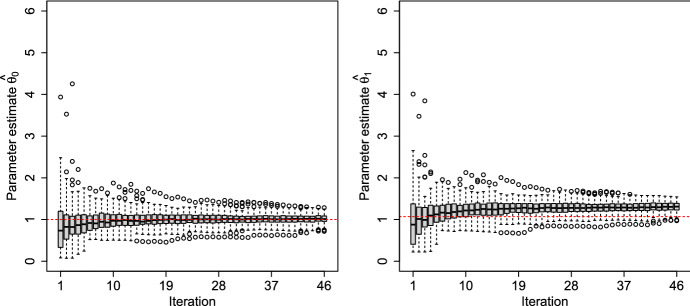
Maximum likelihood estimates of the correlation lengths in Matérn kernels. (Color figure online)

The sequential approach is the only case where the observations ${\textbf{Y}}_n$ corresponding to the previous design points ${\textbf{X}}_n$ are used in the design construction. In our example, we simulate the data according to the assumed model. We use this information to estimate the parameter setting at each step. The (box)plots of the maximum likelihood (ML) estimates ${\hat{\theta }}_0$ and ${\hat{\theta }}_1$ of the inverse correlation lengths $\theta _0$ and $\theta _1$ of $K_{0,\theta }(x,x')$ and $K_{1,\theta }(x,x')$, respectively, are presented in Fig. . This refers to the case where the first kernel, Matérn 3/2, is the data generator. The ${\hat{\theta }}_0$ estimates converge to their null value, $\theta _0=1$, drawn as a red dashed line in the left panel of Fig. 9, as expected due to the consistency of the ML estimator in this case. For the second kernel to be similar to the first one (i.e., less smooth), the ${\hat{\theta }}_1$ estimates have increased (see the right panel). The decrease of the correlation length causes the covariance kernel to drop faster as a function of distance. We defer from presenting the opposite case (where the Matérn 5/2 is the data generator), which is similar.

Apart from the methods applied in Sect. 4, we have considered some other static approaches for discrimination. $D_s$-optimal design is a natural candidate that can be applied in the distance-based fashion. For $D_s$-optimality, we require the general form of the Matérn covariance kernel, which is based on the modified Bessel function of the second kind (denoted by $C_{\nu }$). It is given by

17 $$\begin{aligned} {\textbf{K}}_{\nu }(r)=\dfrac{2^{1-\nu }}{\Gamma (\nu )}\left( \sqrt{2\nu } ~r\theta \right) ^{\nu } C_{\nu }\left( \sqrt{2\nu } ~r\theta \right) . \end{aligned}$$

Smoothness, $\nu$, is considered as the parameter of interest, while the correlation length $\theta$ is assumed as nuisance. The first off-diagonal element in the $2\times 2$ information matrix, associated with the estimation of parameters $\varvec{\theta }=(\theta ,\nu )$, is

18 $$\begin{aligned} M({\textbf{X}}_n,\varvec{\theta })_{12}=\dfrac{1}{2}\textrm{trace}\left\{ {\textbf{K}}_{\nu }^{-1}\dfrac{\partial {\textbf{K}}_{\nu }}{\partial \theta } {\textbf{K}}_{\nu }^{-1}\dfrac{\partial {\textbf{K}}_{\nu }}{\partial \nu } \right\} , \end{aligned}$$

see, e.g., Eq. (6.19) in Müller (2007). The other elements in the information matrix are calculated similarly. We have used the supplementary material of Lee et al. (2018) to compute the partial derivatives of the Matérn covariance kernel. Finally, the $D_s$-criterion is

19 $$\begin{aligned} \Phi _{D_s }= |M({\textbf{X}}_n,\varvec{\theta })|/ |M({\textbf{X}}_n,\varvec{\theta })_{11}|, \end{aligned}$$

where $M({\textbf{X}}_n,\varvec{\theta })_{11}$ is the element of the information matrix corresponding to the nuisance parameter (i.e., in $M({\textbf{X}}_n,\varvec{\theta })_{11}$ both partial derivatives are calculated with respect to $\theta$). In the examples to follow we consider local $D_s$-optimal design; that is, the parameters $\theta$ and $\nu$ are set at given values.

From a Bayesian perspective, models can be discriminated optimally when the difference between the expected entropies of the prior and the posterior model probabilities is maximised. This criterion underlies a famous sequential procedure put forward by Box and Hill (1967) and Hill and Hunter (1969). Since such criteria typically cannot be computed analytically, several bounds were derived. The upper bound proposed by Box and Hill (1967) is equivalent to the symmetric Kullback-Leibler divergence $\Phi _{KL}$. Hoffmann (2017) derives a lower bound based on a lower bound for the Kullback–Leibler divergence between a mixture of two normals, which is given by Eq. (A3) and is denoted by $\Phi _{\Gamma }$. Here, we assume equal prior probabilities. A more detailed account of Bayesian design criteria and their bounds is given in Appendix A.

**Table 1:** Comparison of average hit rates in different methods for the first numerical example

	Average hit rate									
Design size	5	6	7	8	9	10	20	30	40	50
Sequential (4)	0.500	0.535	0.540	0.595	0.570	0.640	0.695	0.715	0.740	0.770
$\phi _A$	0.505	0.500	0.530	0.525	0.505	0.510	0.520	0.535	0.585	0.635
$\phi _B$	0.520	0.545	0.575	0.585	0.615	0.650	0.785	0.875	0.900	0.910
$\phi _{KL}$	0.520	0.545	0.575	0.585	0.615	0.650	0.785	0.870	0.915	0.925
$\Phi _F$	0.580	${\textbf { 0.625}}$	${\textbf {0.620}}$	0.625	0.670	${\textbf {0.715}}$	0.795	${\textbf {0.900}}$	0.925	0.950
$\Phi _1$	0.525	0.520	0.555	0.540	0.550	0.610	0.725	0.890	0.910	0.920
$\Phi _2$	0.525	0.520	0.555	0.540	0.550	0.610	0.715	0.860	0.890	0.910
$\Phi _{KL}$	0.580	${\textbf {0.625}}$	${\textbf {0.620}}$	0.625	0.670	${\textbf {0.715}}$	0.795	0.895	0.925	${\textbf {0.955}}$
$\Phi _{\Gamma }$	${\textbf {0.595}}$	${\textbf {0.625}}$	0.610	${\textbf {0.645}}$	${\textbf {0.675}}$	0.700	0.795	0.895	${\textbf {0.935}}$	0.940
$\Phi _{D_s}$	0.540	0.575	0.590	0.620	0.650	0.675	${\textbf {0.805}}$	0.850	0.855	0.925

Bold numbers indicate the highest average hit rate achieved for each design size

Table collects simulation results for the given example. We have included the sequential procedure (4) as a benchmark for orientation. For all other approaches the true parameter values are used in the covariance kernels. Concerning static (distance-based) designs based on maximisation of $\Phi _F,\Phi _1,\Phi _2,\Phi _{KL},\Phi _\Gamma ,\Phi _{D_s}$, for each design size considered we first built a an incremental design and then used a classical exchange-type algorithm to improve it. These designs are thus not necessarily nested, i.e., ${\textbf{X}}_n\not \subset {\textbf{X}}_{n'}$ for $n<n'$.

Each design of size *n* was then evaluated by generating $N=100$ independent sets of *n* observations generated with the assumed true model, evaluating the likelihood functions for these sets of observations for both models, and then deciding for each set of observations which model has the higher likelihood value. The hit rate is the fraction of sets of observations where the assumed true model has the higher likelihood value. The procedure was repeated by assuming the other model to be the true one. The two hit rates are then averaged and stated in Table 1, which contains the results for all the criteria and design sizes we considered. For the special case of the sequential construction (4), the design path depends on the observations generated at the previously selected design points; that is, unlike for the other criteria, for a given design size *n* each random run produces a different design. To compute the hit rates for a particular *n* we used $N=100$ independent runs of the experiment.

The hit rates reported in Table 1 reflect the discriminatory power of the corresponding designs. One can observe that $\Phi _F$ and as expected $\Phi _{KL}$ are outperforming in terms of hit rates. The Bayesian lower bound criterion $\Phi _{\Gamma }$ is similar to the symmetric $\Phi _{KL}$. The sequential design strategy (4) does not behave as well as the outperforming ones. It is, however, the realistic scenario that one might consider in applications as it does not assume knowledge of the kernel parameters. The effect of this knowledge can thus be partially calibrated for by comparing the first line against the other criteria.

### Optimal design measure for $\phi _p$

Theorem 1 also allows the use of approximate designs as it presents a necessary condition for optimality of the family of criteria $\phi _p,~p>0$. This is more extensively discussed in the previous section. Here we present the numerical results for two specific cases of $p=2$ and $p=10$. To reach a design which might be numerically optimal (or at least nearly optimal), we have applied the Fedorov–Wynn algorithm (Fedorov 1971; Wynn 1970) on a dense regular grid of candidate points.

**Fig. 10:**
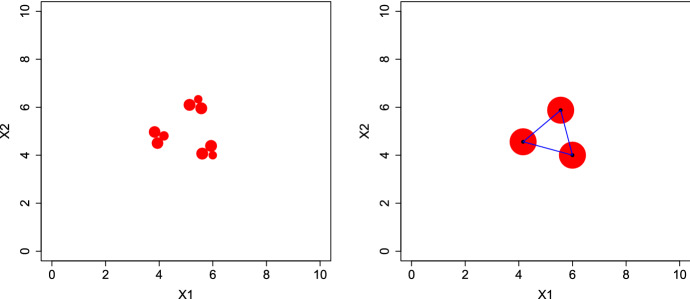
Left: The optimal measure for $\phi _{2}$. Right: The optimal measure for $\phi _{10}$. (Color figure online)

Numerical results show that for very small *p* (e.g., $p=1$) explicit optimal measures are hard to derive. The left panel in Fig.  presents the measure $\xi _2^*$ obtained for $\phi _{2}$. To construct $\xi _2^*$, we have first calculated an optimal design on a dense grid by applying 1000 iterations of the Fedorov–Wynn algorithm (see the comment following Theorem 1); the design measure obtained is supported on 9 grid points. We then applied a continuous optimisation algorithm (library NLopt (Johnson 2021) through its R-interface nloptr) initialised at this 9-point design. The 9 support points of the resulting design measure $\xi _2^*$ are independent of the grid size; they receive unequal weights, proportional to the disk areas on Fig. 10-left. Any translation or rotation of $\xi _2^*$ yields the same value of $\phi _2$.

As the order *p* increases, we eventually reach an optimal measure with only three support points and equal weights. The right panel in Fig. 10 corresponds to the optimal design measure computed for $\phi _{10}$. This has, similarly as before, resulted from application of a continuous optimisation initialised at an optimal 3-point design calculated with the Fedorov–Wynn algorithm on a grid. This optimal design measure $\xi _{10}^*$ has three support points, drawn as blue dots, with equal weights 1/3 represented by the areas of the red disks. The blue line segments between every two locations have length $\Delta \simeq 1.92$, reflecting the ideal interpoint distance (see the right panel of Fig. 8), in agreement with corresponding discussions in Sect. 5. Also here the optimal designs are rotationally and translationally invariant, and thus any design of such type is optimal as long as the design region is large enough to fit it.

## Conclusions

In this paper we have considered the design problem for the discrimination of Gaussian process regression models. This problem differs considerably from the well-treated one in standard regression models and thus offers a multitude of challenges. While the KL-divergence is a straightforward criterion, it comes with the price of being computationally demanding and lacking convenient simplifications such as design measures. We have therefore introduced a family of criteria that allow such a simplification at least in special cases and have investigated its properties. We have also compared the performance of these and other potential criteria on several examples and see that KL-divergence can be effectively replaced by simpler criteria without much loss in efficiency. In particular designs based on the Fréchet-distance between covariance kernels seem to be competitive. Results from the approximate design computations indicate that for classical isotropic kernels, designs with $d+1$ support points placed at the vertices of a simplex of suitable size are optimal for distance-based criteria $\phi _p$ for a large enough *p* when the design region is small enough and are marginally suboptimal otherwise.

As a next step, it would be interesting to investigate the properties of the discrimination designs under parameter uncertainty, for example by considering minimax or Bayesian designs.

A referee has indicated that our techniques could be used for discriminating the intricately convoluted covariances stemming from deep Gaussian processes (as defined in Damianou and Lawrence (2013)) from more conventional ones. This is an interesting issue of high relevance for computer simulation experiments that certainly needs to be explored in the future.

## Appendix A: Notes on Box–Hill–Hunter Bayesian criteria for model discrimination between Gaussian random fields

Chapter 5 of Hoffmann (2017) contains an overview of Bayesian design criteria for model discrimination and some useful bounds on them. We assume there are *M* models $m_0,\ldots ,m_{M-1}$. The most common Bayesian design criterion for model discrimination has the following form:

A1 $$\begin{aligned} \Phi _{\Lambda }({\textbf{X}}_k){} & {} = -\sum _{i=0}^{M-1} p(m_i) \log (p(m_i)) \nonumber \\{} & {} \quad + \int _{{\textbf{Y}}_k \in {\mathcal {Y}}} p({\textbf{Y}}_k) \sum _{i=0}^{M-1} p(m_i \arrowvert {\textbf{Y}}_k) \log (p(m_i \arrowvert {\textbf{Y}}_k)) \, \textrm{d}{\textbf{Y}}_k, \end{aligned}$$

where the data ${\textbf{Y}}_k = (Y_1(x_1),\ldots ,Y_k(x_k))^\top$ are observed at the design ${\textbf{X}}_k = (x_1,\ldots ,x_k)$, $p(m_i)$ denotes the prior and $p(m_i \arrowvert {\textbf{Y}}_k)$ the posterior model probability of model $m_i$ and $p({\textbf{Y}}_k)$ is the marginal distribution of ${\textbf{Y}}_k$ with respect to the models. Hence, this criterion is the (expected) difference of the model entropy and the conditional model entropy (conditional on the observations). The posterior model probability $p(m_i \arrowvert {\textbf{Y}}_k)$ is defined by

$$\begin{aligned} p(m_i \arrowvert {\textbf{Y}}_k) \propto p({\textbf{Y}}_k \arrowvert m_i) p(m_i), \end{aligned}$$

where $p({\textbf{Y}}_k \arrowvert m_i)$ is the likelihood of model $m_i$ (marginalised over the parameters), and $p({\textbf{Y}}_k)$ is given by

$$\begin{aligned} p({\textbf{Y}}_k) = \sum _{i=0}^{M-1} p({\textbf{Y}}_k \arrowvert m_i) p(m_i). \end{aligned}$$

The first term in (A1) does not depend on the design and can therefore be ignored.

A common alternative formulation of criterion (A1) is the one adopted by Box and Hill (1967) and Hill and Hunter (1969), which will henceforth be called Box-Hill-Hunter (BHH) criterion:

A2 $$\begin{aligned} \Phi _{\Lambda }({\textbf{X}}_k) = \sum _{i=0}^{M-1} p(m_i) \int _{{\textbf{Y}}_k \in {\mathcal {Y}}} p({\textbf{Y}}_k \arrowvert m_i) \log \left( \frac{p({\textbf{Y}}_k \arrowvert m_i)}{p({\textbf{Y}}_k)} \right) \, \textrm{d}{\textbf{Y}}_k. \end{aligned}$$

In our case, if we assume point priors for the kernel parameters, we have

$$\begin{aligned} p({\textbf{Y}}_k \arrowvert m_i) = \varphi ({\textbf{Y}}_k \arrowvert \varvec{\eta }_{k,i}, {\textbf{K}}_{k,i}), \end{aligned}$$

where $\varvec{\eta }_{k,i} = (\eta _{1,i}(x_1),\ldots ,\eta _{k,i}(x_k))^\top$ is the mean vector of model *i* at design ${\textbf{X}}_k$, ${\textbf{K}}_{k,i}$ is the $k \times k$ kernel matrix of model *i* with elements given by $\{{\textbf{K}}_{k,i}\}_{j,l} = K_i(x_j, x_l)$, and $\varphi (\cdot \arrowvert \varvec{\eta },{\textbf{K}})$ is the normal pdf with mean vector $\varvec{\eta }$ and variance-covariance matrix ${\textbf{K}}$.

For example, for a static design involving *n* design points, we set $k = n$ and assume that $\varvec{\eta }_{n,i} = {\textbf{0}}$ for each design ${\textbf{X}}_n$. The model probabilities $p(m_i)$ would just be the prior model probabilities before having collected any observations.

In a sequential design setting, where *n* observations ${\textbf{Y}}_n$ have already been observed at locations ${\textbf{X}}_n$ and we want to find the optimal design point *x* where to collect our next observation, we have $k=1$ and set $\varvec{\eta }_{k,i}$ to the conditional mean ${\widehat{\eta }}_{n,i}(x) = {\textbf{k}}_{n,i}(x)^\top {\textbf{K}}_{n,i}^{-1} {\textbf{Y}}_n$ and ${\textbf{K}}_{k,i}$ to the conditional variance $\rho _{n,i}^2(x) = K_i(x,x) - {\textbf{k}}_{n,i}(x)^\top {\textbf{K}}_{n,i}^{-1} \, {\textbf{k}}_{n,i}(x)$, where ${\textbf{k}}_{n,i}(x)^\top = (K_i(x,x_1),\ldots ,K_i(x,x_n))$, see Sect. 3.1. The prior model probabilities would have to be set to the posterior model probabilities given the already observed data:

$$\begin{aligned} p(m_i) = p(m_i \arrowvert {\textbf{Y}}_n) \propto \varphi ({\textbf{Y}}_n \arrowvert \, {\textbf{0}}, {\textbf{K}}_{n,i}) \, p(m_i). \end{aligned}$$

It follows that $p({\textbf{Y}}_k)$ is a mixture of normal distributions. The criterion representations (A1) and (A2) cannot be computed directly. However, several bounds have been developed for the criterion, the most famous being the classic upper bound derived by Box and Hill (1967).

### Appendix A.1: Upper bound

The upper bound has the following form (see also Hoffmann (2017, Thm. 5.2, p. 168)):

$$\begin{aligned} \Phi _{U}({\textbf{X}}_k) = \frac{1}{2} \sum _{i=0}^{M-1} \sum _{j=0}^{M-1} p(m_i) p(m_j) \left\{ \left\| \varvec{\eta }_{k,i}-\varvec{\eta }_{k,j}\right\| ^2_{{\textbf{K}}_{k,j}^{-1}} + \textrm{trace}\left( {\textbf{K}}_{k,i} {\textbf{K}}_{k,j}^{-1}\right) - n \right\} . \end{aligned}$$

For $M=2$, the formula simplifies to

$$\begin{aligned} \Phi _{U}({\textbf{X}}_k){} & {} = \frac{1}{2} p(m_0) p(m_1) \biggl \{ \left\| \varvec{\eta }_{k,0}-\varvec{\eta }_{k,1}\right\| ^2_{{\textbf{K}}_{k,0}^{-1}} + \left\| \varvec{\eta }_{k,0}-\varvec{\eta }_{k,1}\right\| ^2_{{\textbf{K}}_{k,1}^{-1}} \\{} & {} \quad + \textrm{trace}\left( {\textbf{K}}_{k,0} {\textbf{K}}_{k,1}^{-1}\right) + \textrm{trace}\left( {\textbf{K}}_{k,1} {\textbf{K}}_{k,0}^{-1}\right) - 2n \biggr \}. \end{aligned}$$

This is equivalent to the symmetric Kullback–Leibler divergence that we use as the criterion $\Phi _{KL}$ (with $p(m_0)=p(m_1)=1/2$ and $\varvec{\eta }_{k,0}=\varvec{\eta }_{k,1}={\textbf{0}}$).

### Appendix A.2: Lower bound

Hershey and Olsen (2007, Sect. 7) derive a lower bound for the Kullback–Leibler divergence between a mixture of two normals, see also Hoffmann (2017, Thm. 5.4 and Cor. 5.5, pp. 173–174). This result is then used by Hoffmann (2017) to find a lower bound for the BHH criterion $\Phi _{\Lambda }({\textbf{X}}_k)$ (Hoffmann 2017, Thm. 5.9, p. 178). This lower bound is given by

$$\begin{aligned} \Phi _{\Gamma }({\textbf{X}}_k) = -\sum _{i=0}^{M-1} p(m_i) \log \left\{ \sum _{j=0}^{M-1} p(m_j) \exp \left( -\frac{1}{2} \varvec{\Gamma }({\textbf{X}}_k)_{ij} \right) \right\} , \end{aligned}$$

where

$$\begin{aligned} \varvec{\Gamma }({\textbf{X}}_k)_{ij} = \left\| \varvec{\eta }_{k,i}-\varvec{\eta }_{k,j}\right\| ^2_{{\textbf{K}}_{k,j}^{-1}} + \textrm{trace}\left( {\textbf{K}}_{k,i} {\textbf{K}}_{k,j}^{-1}\right) - \log \det \left( {\textbf{K}}_{k,i} {\textbf{K}}_{k,j}^{-1}\right) - n. \end{aligned}$$

For $M=2$, which is the relevant case for our setup, we get

A3 $$\begin{aligned} \Phi _{\Gamma }({\textbf{X}}_k){} & {} = -p(m_0) \log \biggl \{ p(m_0) \nonumber \\{} & {} \quad + p(m_1) \exp \biggl ( -\frac{1}{2} \biggl [ \left\| \varvec{\eta }_{k,0}-\varvec{\eta }_{k,1}\right\| ^2_{{\textbf{K}}_{k,1}^{-1}} + \textrm{trace}\left( {\textbf{K}}_{k,0} {\textbf{K}}_{k,1}^{-1}\right) \nonumber \\{} & {} \quad - \log \det \left( {\textbf{K}}_{k,0} {\textbf{K}}_{k,1}^{-1}\right) -n \biggr ] \biggr ) \biggr \} \nonumber \\{} & {} \quad - p(m_1) \log \biggl \{ p(m_1) \nonumber \\{} & {} \quad + p(m_0) \exp \biggl ( -\frac{1}{2} \biggl [ \left\| \varvec{\eta }_{k,0}-\varvec{\eta }_{k,1}\right\| ^2_{{\textbf{K}}_{k,0}^{-1}} + \textrm{trace}\left( {\textbf{K}}_{k,1} {\textbf{K}}_{k,0}^{-1}\right) \nonumber \\{} & {} \quad - \log \det \left( {\textbf{K}}_{k,1} {\textbf{K}}_{k,0}^{-1}\right) -n \biggr ] \biggr ) \biggr \} \end{aligned}$$

where $\varphi _i(\cdot ) = \varphi (\cdot \arrowvert \varvec{\eta }_{k,i},{\textbf{K}}_{k,i})$, which we are also using to compute designs in Sect. 6.1 (again with $p(m_0)=p(m_1)=1/2$ and $\varvec{\eta }_{k,0}=\varvec{\eta }_{k,1}={\textbf{0}}$).
